# Quality, reliability, and dissemination of lung cancer information on short-video platforms in China: a cross-platform content analysis of TikTok, Kwai, and Rednote

**DOI:** 10.3389/fpubh.2025.1683561

**Published:** 2025-12-01

**Authors:** Xiaoran Zheng, Qiankuan Li, Lu Jin, Kaihang Shi, Mengqi Deng

**Affiliations:** 1 Handan Fukang Hospital, Handan, China; 2 Zhongshan Institute for Drug Discovery, Shanghai Institute of Materia Medica, Chinese Academy of Sciences, Zhongshan, Guangdong, China; 3 Department of Dermatology and Venereology, Affiliated Hospital of Chengde Medical University, Chengde, Hebei, China; 4 Beijing Key Laboratory for Tumor Invasion and Metastasis, Department of Biochemistry and Molecular Biology, Capital Medical University, Beijing, China

**Keywords:** lung cancer, digital health, social media, health communication, short-video, health information quality

## Abstract

**Background:**

Short-video platforms have become major sources of health information in China, influencing public awareness and health behavior. However, the quality and dissemination patterns of lung cancer–related content across different platforms remain unclear. This study aimed to evaluate the informational quality, reliability, and engagement patterns of lung cancer short videos on three leading Chinese platforms.

**Methods:**

We conducted a comprehensive cross-sectional content analysis of 1,288 lung cancer-related videos retrieved from TikTok, Kwai and Rednote. Video quality was systematically evaluated using a multidimensional toolkit, including the Journal of the American Medical Association (JAMA) benchmark criteria, Global Quality Scale (GQS), modified DISCERN (mDISCERN), and the Patient Education Materials Assessment Tool (PEMAT-U/A). We analyzed heterogeneity and correlations of quality and engagement metrics (likes, comments, shares, collections) across platforms, creator types, content themes, and presentation formats.

**Results:**

Overall information quality was suboptimal (Median JAMA = 2; Median GQS = 3). Significant heterogeneity (*p* < 0.001) was found, with TikTok demonstrating the highest quality, while Kwai exhibited the lowest quality but high engagement. Videos by physicians and news agencies demonstrated significantly higher reliability, understandability, and actionability than those by non-professional creators (*p* < 0.001). Disease knowledge videos—particularly those focusing on prevention, definitions, and risk factors—exhibited superior quality compared to personal experience or metastasis-related content. Expert monolog videos were the most common and effective presentation format. Engagement did not align linearly with quality. Patient vlogs and metastasis-related videos achieved higher interaction rates despite lower accuracy, indicating a “quality–engagement paradox.” Weak-to-moderate positive correlations were found between GQS and engagement, while PEMAT-A was negatively correlated with likes and comments.

**Conclusion:**

Marked disparities in the quality and dissemination of lung cancer–related short videos exist across Chinese platforms. Professional, evidence-based content enhances reliability, whereas emotional and visually driven content drives engagement. Strengthening algorithmic governance, metadata transparency, and expert involvement—alongside audience-centered, evidence-informed communication—may enhance the educational value and public health impact of short-video platforms.

## 1 Introduction

Lung cancer remains the leading cause of cancer incidence and mortality worldwide, with an estimated 2.5 million new cases and 1.8 million deaths in 2022 (1). China bears a disproportionately high burden, accounting for a substantial proportion (30–50%) of global cases (2). The disease’s growing prevalence is driven by population aging, industrialization, and tobacco exposure, while emerging evidence highlights additional risks from air pollution and occupational hazards (3).

Despite advances in targeted and immune therapies, approximately half of patients are diagnosed at advanced stages, and overall prognosis remains poor (4). Low-dose computed tomography (LDCT) screening effectively reduces mortality (5). However, its wider adoption has increased detection of indolent lesions, raising concerns about overdiagnosis and overtreatment (6). Balancing delayed diagnosis and excessive intervention therefore requires more than technological improvement. The public should be well informed about the risk factors, early symptoms, treatment options, and potential benefits and harms of screening. Enhancing public understanding of risk factors, early symptoms, screening benefits and harms, and treatment options is essential for informed decision-making (7).

In parallel, short-video platforms have rapidly become major sources of health information in China (8). Platforms such as TikTok (Douyin in China market), Kwai (Kuaishou), and Rednote (Xiaohongshu) have each reported several hundred million active users by 2024, illustrating their enormous public reach and growing role in health communication (9–11). Short-form videos can increase attention, comprehension, and behavioral intention, providing an efficient way to public health education (12). However, the same open and entertainment-oriented ecology facilitates misinformation, incomplete narratives, and unverified medical claims (13, 14). Existing analyses have revealed frequent absence of source attribution, limited professional oversight, and weak adherence to evidence-based standards, leaving the reliability of lung-cancer-related content uncertain (12).

We employed a suite of standardized tools—Journal of the American Medical Association (JAMA) benchmark criteria, Global Quality Scale (GQS), modified DISCERN (mDISCERN), and the Patient Education Materials Assessment Tool (PEMAT-U/A)—to conduct a comprehensive, multidimensional assessment of online health information quality. Nevertheless, these instruments have rarely been applied in a unified, cross-platform manner to short-video ecosystems, and how informational quality interacts with dissemination remains largely unexplored. Therefore, this study systematically analyzed lung-cancer-related short videos from three major Chinese platforms, using validated instruments. By integrating quality and engagement indicators, we aimed to (i) compare informational performance across platforms, (ii) identify topic-specific quality patterns, and (iii) explore relationships between video quality and dissemination. These findings provide evidence-based insights for improving the reliability and educational value of digital health communication.

## 2 Methods

### 2.1 Study design

This study employed a cross-sectional content analysis of publicly available short videos on major Chinese platforms, without involving human participants or identifiable personal information.

### 2.2 Data retrieval and collection

Video retrieval was conducted between 22 February and 7 March 2025 across major platforms: TikTok, Kwai, Rednote. Videos were ranked according to each platform’s default algorithm. To minimize algorithmic bias, a newly registered account was used for all searches, with browsing histories cleared, fixed IP addresses maintained, and no personal or mobile information linked.

Three standardized keywords— “lung cancer,” “primary bronchogenic carcinoma” and “primary bronchogenic lung cancer”—were used for searching. Previous research has shown that most audiences primarily focus on the initial search results (15, 16). The first 200 videos returned per keyword were collected from each platform (total = 1,800). After removing duplicates and applying predefined inclusion and exclusion criteria, a final sample of 1,288 videos was retained for analysis, distributed as follows: 454 (35.3%) from TikTok, 428 (33.2%) from Kwai, and 406 (31.5%) from Rednote. Detailed distributions are presented in Supplementary Table S1.

The inclusion criteria were: (1) videos primarily focused on lung cancer and (2) videos presented in Mandarin or English.

The exclusion criteria were: (1) duplicate videos, (2) promotional or commercial content, and (3) materials unrelated to lung cancer.

For each eligible video, the following variables were extracted: title, duration, upload date, number of likes, comments, collections, shares, uploader type, follower count, content theme, and presentation format. This protocol simulated non-personalized retrieval conditions to enhance reproducibility and comparability. Detailed distributions are provided in Supplementary Table S1.

### 2.3 Video quality assessment

Video quality was independently evaluated by two trained researchers (Z-XR and L-QK) using four validated instruments. JAMA benchmarks (range = 0–4) are widely recognized as a robust framework for assessing the reliability of online health information (17). GQS uses a five-point Likert scale to measure overall content quality (17, 18). The mDISCERN contains five items evaluating the reliability and quality of online health materials (19, 20). These metrics are commonly employed in similar studies (21–23). The PEMAT developed by the Agency for Healthcare Research and Quality, evaluates health educational materials by assessing both PEMAT-U and PEMAT-A (24). Detailed scoring criteria are available in Supplementary Tables S2–S4.

Before formal scoring, both raters jointly reviewed 20 training videos to ensure consistent interpretation. Each video was rated three times—twice by Z-XR and once by L-QK. Inter-rater reliability was assessed using weighted kappa (κ_w_) for ordinal scales (JAMA, GQS, and DISCERN) and intraclass correlation coefficients (ICC) for continuous measures (PEMAT-U and PEMAT-A). Interpretations followed established thresholds: ≤0.40 = poor, 0.41–0.60 = moderate, 0.61–0.80 = substantial, >0.80 = excellent. Final scores were determined by consensus (25).

Inter-rater reliability demonstrated strong consistency across all measures: JAMA (*κ*_w_ = 0.88, 95% CI [0.85–0.90]), GQS (*κ*_w_ = 0.82, 95% CI [0.79–0.85]), mDISCERN (*κ*_w_ = 0.86, 95% CI [0.83–0.88]), PEMAT-U (ICC = 0.93, 95% CI [0.92–0.94]), and PEMAT-A (ICC = 0.87, 95% CI [0.86–0.88]). Intra-rater reliability was also excellent: JAMA (*κ*_w_ = 0.98), GQS (*κ*_w_ = 0.96), mDISCERN (*κ*_w_ = 0.96), PEMAT-U (ICC = 0.98), and PEMAT-A (ICC = 0.94).

### 2.4 Statistical analysis

Data procedures and visualization were performed using SPSS v22.0 (IBM Corp., Armonk, NY), GraphPad Prism v10.1.2 (Dotmatics, Boston, MA), and OriginPro 2021 v9.8.0.204 (OriginLab Corp., Northampton, MA) and R 4.5.1 (R foundation for statistical computing, Vienna, Austria). Data normality was tested using the Kolmogorov–Smirnov test. Continuous variables are presented as median (interquartile range [IQR]), and categorical variables as counts and percentages. For group comparisons, the Kruskal–Wallis test was applied, followed by Dunn’s *post hoc* test when appropriate. Spearman’s rank correlation assessed associations among variables. Statistical significance was set at *p* < 0.05.

### 2.5 Ethical considerations

This study analyzed only publicly available videos from TikTok, Kwai, and Rednote, without collecting personal identifiers or private information. The Handan Fukang Hospital Ethics Committee reviewed the protocol and granted formal ethical-review exemption, confirming compliance with institutional and national guidelines for studies not involving human subjects. All data collection procedures adhered to platform Terms of Service and the *Declaration of Helsinki* principles for responsible use of publicly available digital content.

## 3 Results

**Figure 1:**
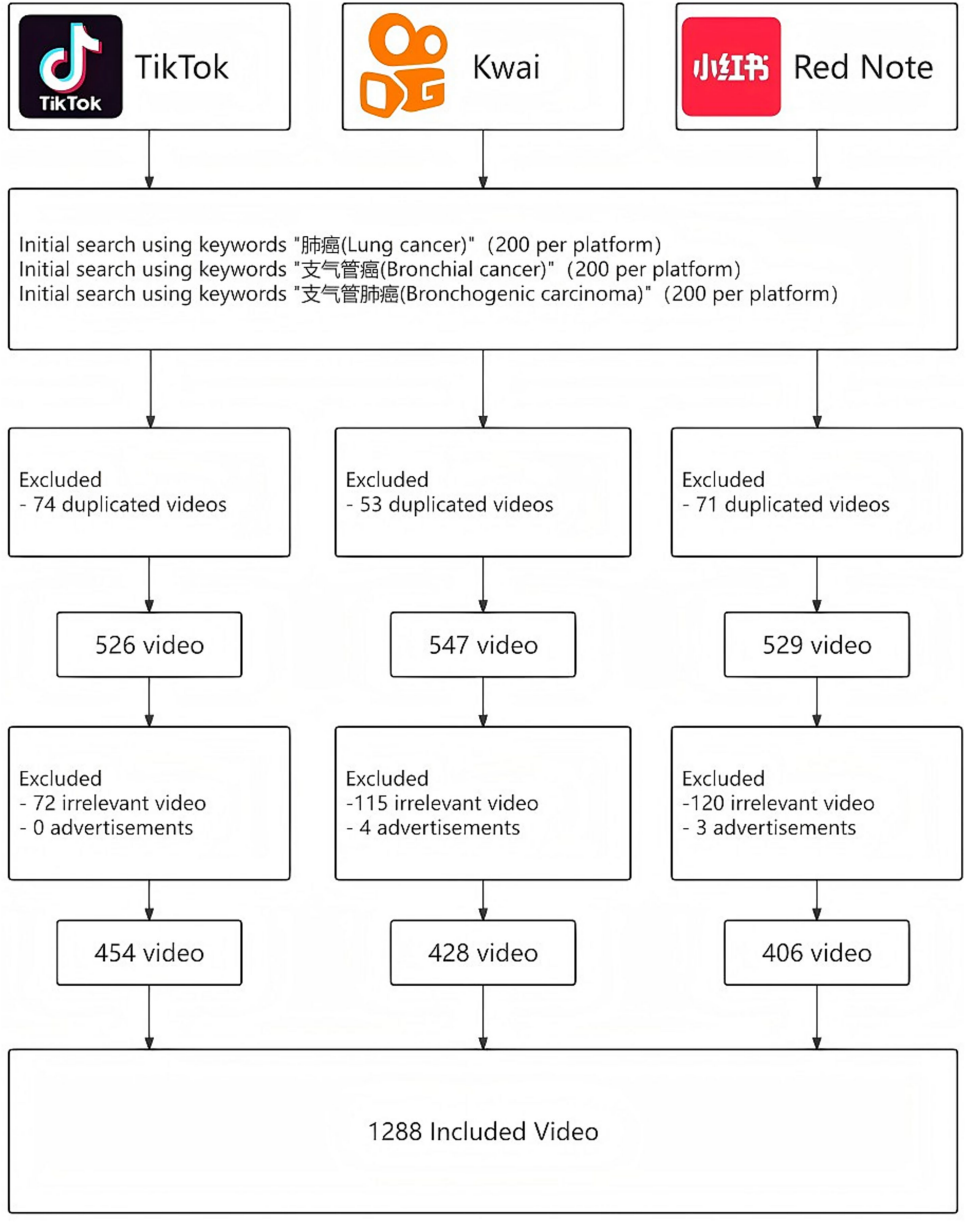
Flowchart of the study.

After exclusions, 1,288 lung cancer–related videos were included in the analysis: 454 (35.3%) from TikTok, 428 (33.2%) from Kwai, and 406 (31.5%) from Rednote (Figure 1).

### 3.1 Characteristics of videos and inter-platform differences

**Figure 2:**
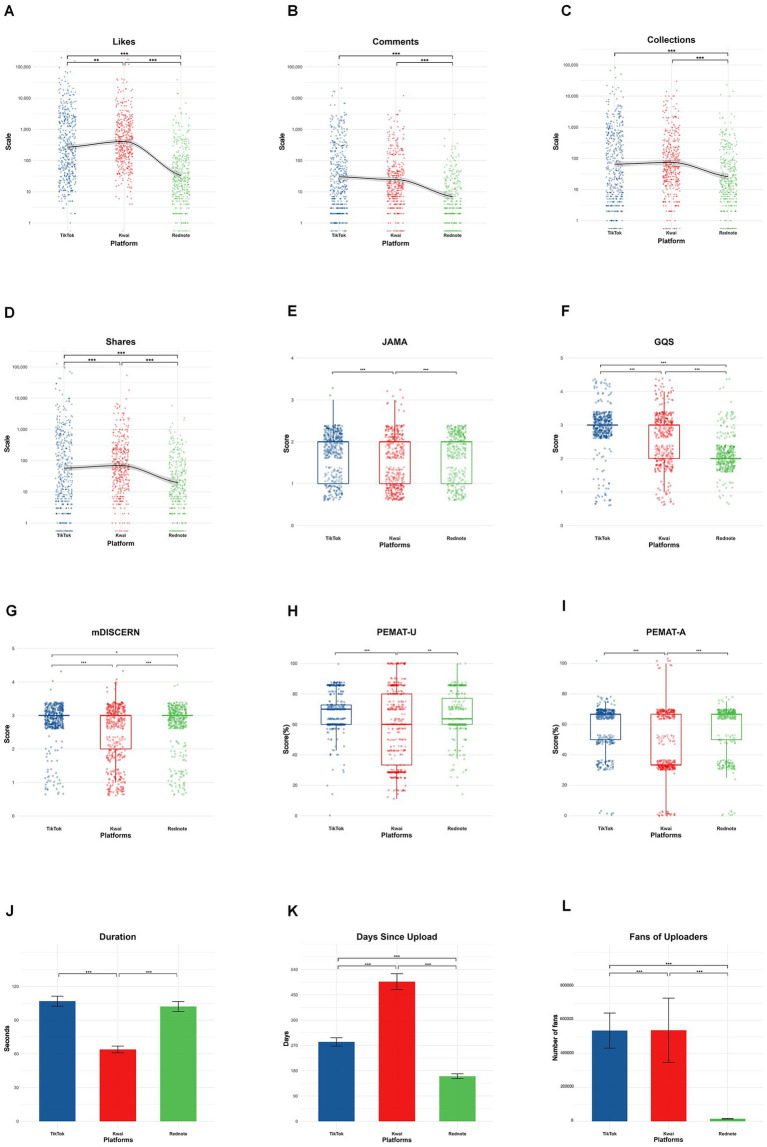
Comparison of engagement indicators, quality scores, and general characteristics of lung cancer–related videos across three short-video platforms. **(A–D)** Engagement metrics: Likes, Comments, Collections, and Shares. **(E–I)** Quality assessment scores: Journal of the American Medical Association (JAMA) score, Global Quality Scale (GQS) score, modified DISCERN (mDISCERN) score, and Patient Education Materials Assessment Tool—Understandability (PEMAT-U) and Actionability (PEMAT-A). **(J–L)** General characteristics: Duration, Days since upload, and Number of fans of uploaders. Data are presented as scatterplots **(A–D)**, boxplots **(E–I)**, or bar graphs with error bars (J-L). *p < 0.05, **p < 0.01, ***p < 0.001 indicate significance; “ns” denotes non-significant differences.

General characteristics are summarized in Table 1 and Figure 2. The median video length was 71 s (IQR: 37–118). Videos from TikTok and Rednote were significantly longer than those on Kwai (*p* < 0.001). TikTok and Kwai received similar numbers of comments and collections, both higher than Rednote (*p* < 0.001). Kwai achieved the highest numbers of likes, shares, Fans of video uploaders, and days since upload compared with TikTok (*p* < 0.01–0.001), whereas TikTok outperformed Rednote across these indicators (*η^2^* = 0.17–0.45, 95% CI [0.10, 0.51], *p* < 0.001).

**Table 1:** Characteristics and scoring results of videos concerning lung cancer.

Parameters	Total (*N* = 1,288)	TikTok (*n* = 454)	Kwai (*n* = 428)	Rednote (*n* = 406)	*p*-value
Duration (seconds), median (IQR)	71 (37–118)	81.5 (52–135.25)	53 (17.25–91)	74.5 (43.75–127)	<0.001
Likes, median (IQR)	145.5 (26.25–806.5)	223 (40–1942.25)	381.5 (131–1306.75)	25 (7–103.75)	<0.001
Comments, median (IQR)	10 (2–46)	17 (3–153.5)	16 (5.25–50)	2 (0–11.25)	<0.001
Collections, median (IQR)	34 (6–215)	42 (5–530.25)	54.5 (18–199.5)	15 (4–69)	<0.001
Shares, median (IQR)	26 (5–167.75)	22 (3–365.25)	62 (17–223)	10.5 (2–45.25)	<0.001
Days since upload, median (IQR)	167 (63.25–379.75)	170.5 (63.75–383.75)	263 (102.5–610.25)	106.5 (32–234.25)	<0.001
Fans of video uploaders, median (IQR)	17133.5 (2306.5–104,216)	26,257 (2561.75–169,452)	55429.5 (16974.75–234665.25)	3751.5 (627.5–14913.25)	<0.001
JAMA^a^ score, median (IQR)	2 (1–2)	2 (1–2)	2 (1–2)	2 (1–2)	<0.001
GQS^b^ score, median (IQR)	3 (2–3)	3 (3–3)	3 (2–3)	2 (2–2)	<0.001
mDISCERN score, median (IQR)	3 (3–3)	3 (3–3)	3 (2–3)	3 (3–3)	<0.001
PEMAT-U, median (IQR)	63.64% (60–77.78%)	70% (60–72.73%)	60% (33.33–80%)	60% (60–80%)	<0.001
PEMAT-A, median (IQR)	66.67% (50–66.67%)	66.67% (66.67–66.67%)	33.33% (33.33–66.67%)	66.67% (66.75–66.67%)	<0.001

^a^JAMA, Journal of American Medical Association.

^b^GQS: Global Quality Scale.

Overall informational quality remained limited (Table 1; Figure 2; Supplementary Table S6). The median JAMA score was 2, with 37% (*n* = 476) scoring ≤ 1. The median GQS was 3, with only 3.1% (*n* = 40) reaching high quality (≥4). Median mDISCERN was; highly reliable content (≥4) occurred in 1.0% (*n* = 13). Median PEMAT-U and PEMAT-A scores were 63.6 and 66.7%, respectively; roughly half exceeded 60%, but <1% achieved ≥ 80% (*n* = 10).

Significant platform-specific variation was observed. TikTok achieved the highest overall quality, followed by Rednote and Kwai. Moderate-to-high JAMA (≥2) and mDISCERN (≥3) scores were most frequent on TikTok, whereas Kwai contained the largest proportion of low-quality videos (JAMA = 1 in 49.8%; GQS < 3 in 42.3%; mDISCERN ≤ 2 in 40.4%).

Comparative analyses confirmed these findings: TikTok and Rednote obtained significantly higher JAMA, PEMAT-U, and PEMAT-A scores than Kwai (all *p* < 0.001). TikTok also exceeded Kwai in GQS and mDISCERN (*p* < 0.001), while Rednote ranked between them (*p* < 0.001–0.01).

### 3.2 Video sources, content, and presentation

**Figure 3:**
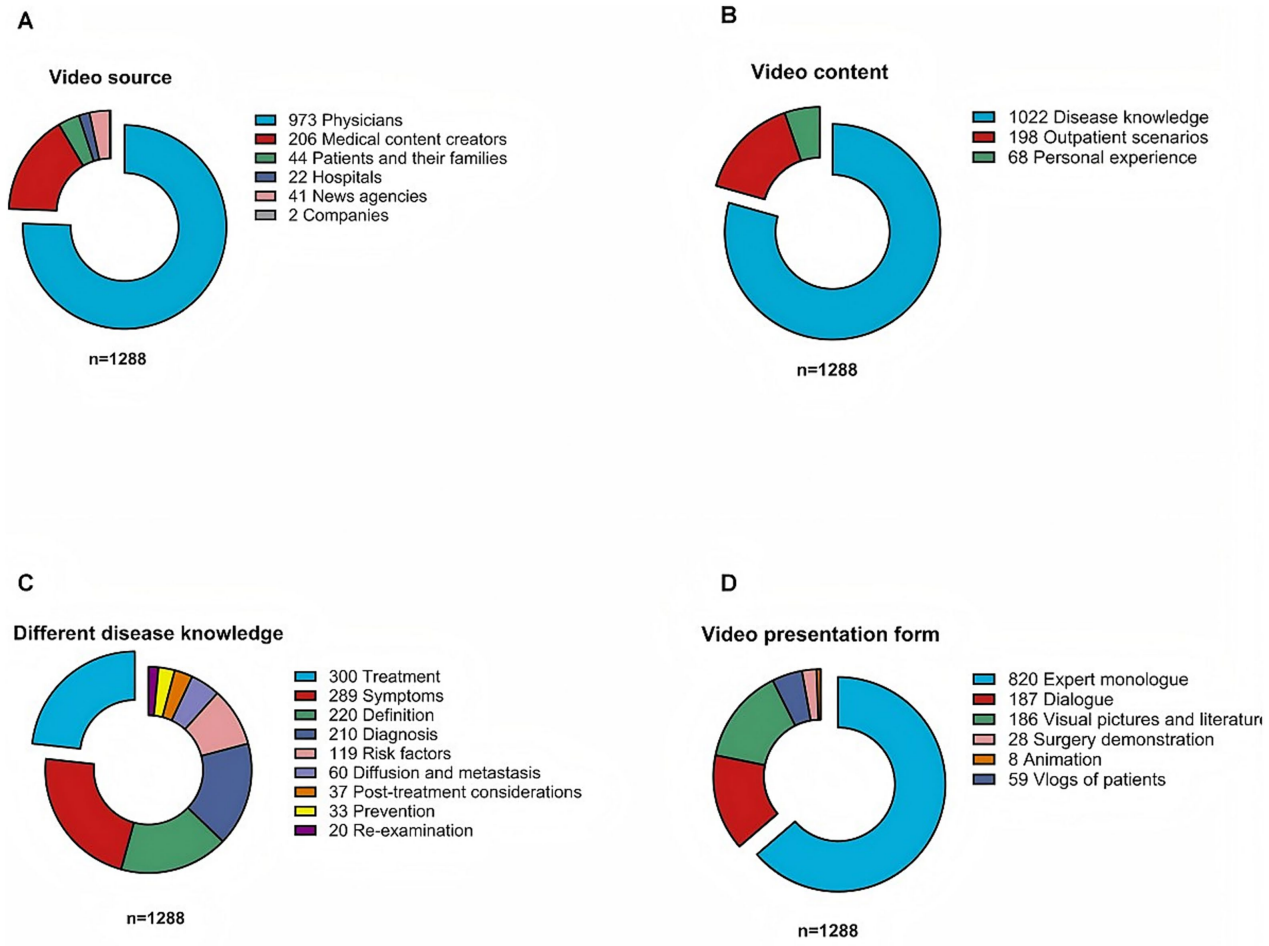
The origins and subject matter of videos pertaining to lung cancer. **(A)** Video sources: The majority of these recordings were published by medical professionals (*n* = 973, 75.5%), followed by medical content creators (*n* = 206, 16.0%), patients and their families (*n* = 44, 3.4%), hospitals (*n* = 42, 3.3%), news agencies (*n* = 21, 1.6%), and companies (*n* = 2, 0.2%). **(B)** Video content categories: The majority of videos focused on disease knowledge (*n* = 1,022, 79.3%), while others featured outpatient scenarios (*n* = 198, 15.4%) or personal experiences (*n* = 68, 5.3%). **(C)** Subcategories of disease knowledge: The content included treatment (*n* = 300), symptoms (*n* = 289), definitions (*n* = 220), diagnosis (*n* = 119), risk factors (*n* = 110), recurrence and metastasis (*n* = 69), post-treatment considerations (*n* = 37), prevention (*n* = 33), and re-examination (*n* = 20). **(D)** Video presentation formats: Expert monolog was the most common form (*n* = 820, 63.7%), followed by dialog (*n* = 187, 14.5%), visual pictures and literature (*n* = 186, 14.4%), surgery demonstrations (*n* = 82, 6.4%), animation (*n* = 8, 0.6%), and patient vlogs (*n* = 59, 4.6%).

**Figure 4:**
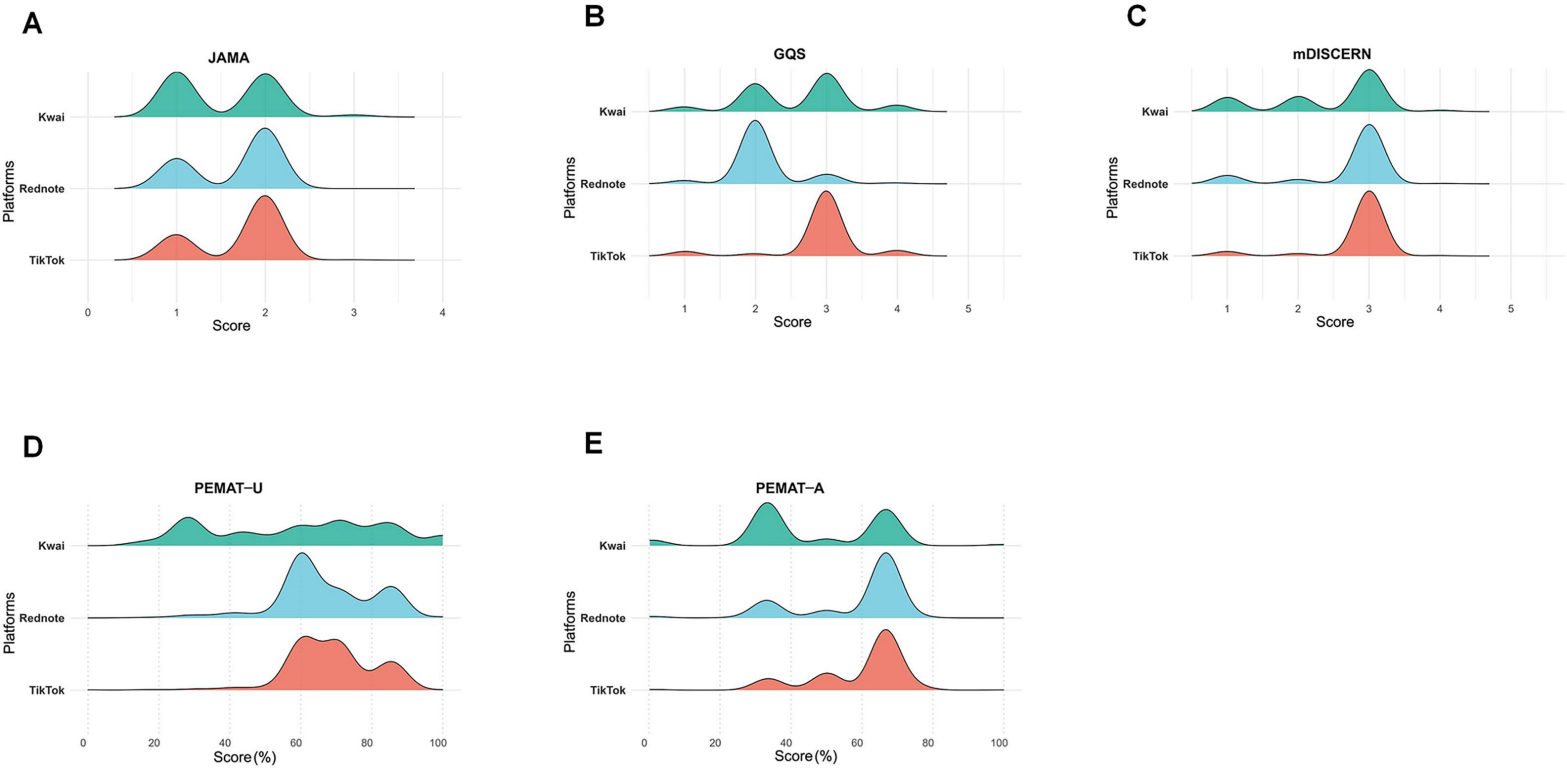
Distribution of quality assessment scores of lung cancer–related videos across three short-video platforms, visualized using ridgeline plots. **(A)** The JAMA score; **(B)** the GQS score; **(C)** the mDISCERN score; **(D)** PEMAT-U; and **(E)** PEMAT-A. Higher peaks indicate greater concentration of videos at corresponding score ranges, illustrating the variation in video quality profiles among platforms.

As shown in Figures 3, 4 and Supplementary Tables S5, S6, source composition, content themes, and presentation forms differed significantly across platforms (*η^2^* = 0.01–0.12, 95% CI [0.002, 0.18], *p* < 0.01–0.001). TikTok and Rednote were dominated by physician-generated content (85.0% each), whereas Kwai relied more on non-professional medical creators (34.4%).

Disease knowledge videos comprised 79.4% of all samples, especially on TikTok (83.5%). Rednote featured more outpatient-scene videos (20.2%) than TikTok (12.6%) or Kwai (13.8%). Platform preferences were distinct: TikTok and Kwai emphasized symptom descriptions (22.0 and 30.1%, respectively), while Rednote prioritized treatment strategies (33.3%).

Presentation forms also varied significantly (*p* = 0.002). Expert monologs were the most common (63.7%), most prevalent on TikTok (69.6%). Kwai contained more visual pictures and literature (21.7%) than TikTok (12.3%) or Rednote (9.1%), whereas Rednote had more dialog videos (19.2%). Animated videos were rare overall (0.6%), found only on Kwai (*n* = 7) and Rednote (*n* = 1).

### 3.3 Determinants of video quality

**Figure 5:**
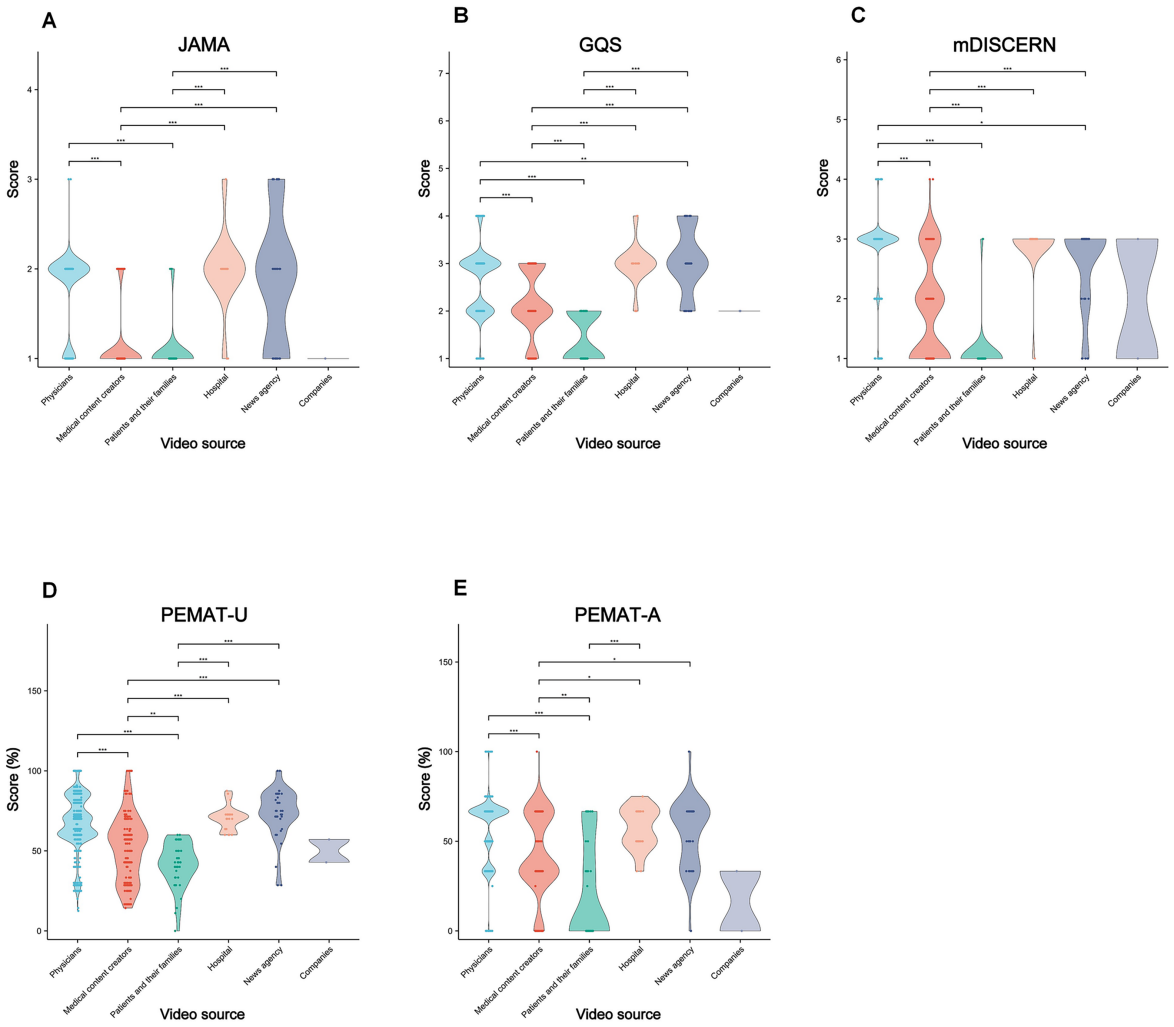
Comparative quality assessment of lung cancer short-videos across sources. Violin plots showing quality evaluation of lung cancer short-videos from the various. **(A)** The JAMA score; **(B)** the GQS score; **(C)** the mDISCERN score; **(D)** PEMAT-U; and **(E)** PEMAT-A. *p < 0.05, **p < 0.01, ***p < 0.001, ns, non-significant.

**Figure 6:**
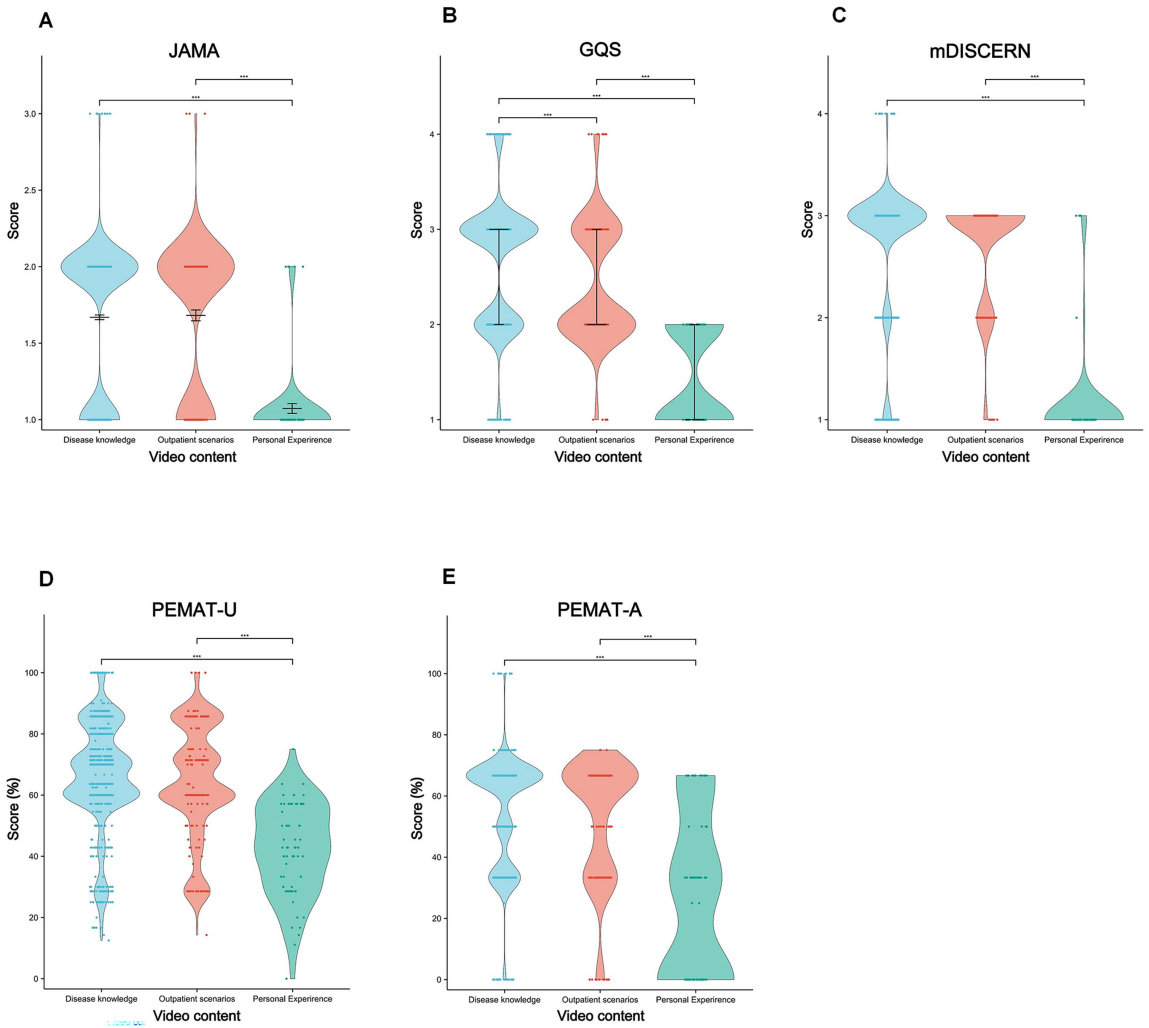
Comparative quality assessment of lung cancer short-videos with different contents. Violin plots showing quality evaluation of lung cancer short videos featuring diverse themes. **(A)** The JAMA score; **(B)** the GQS score; **(C)** the mDISCERN score; **(D)** PEMAT-U; and **(E)** PEMAT-A. *p < 0.05, **p < 0.01, ***p < 0.001, ns, non-significant.

**Figure 7:**
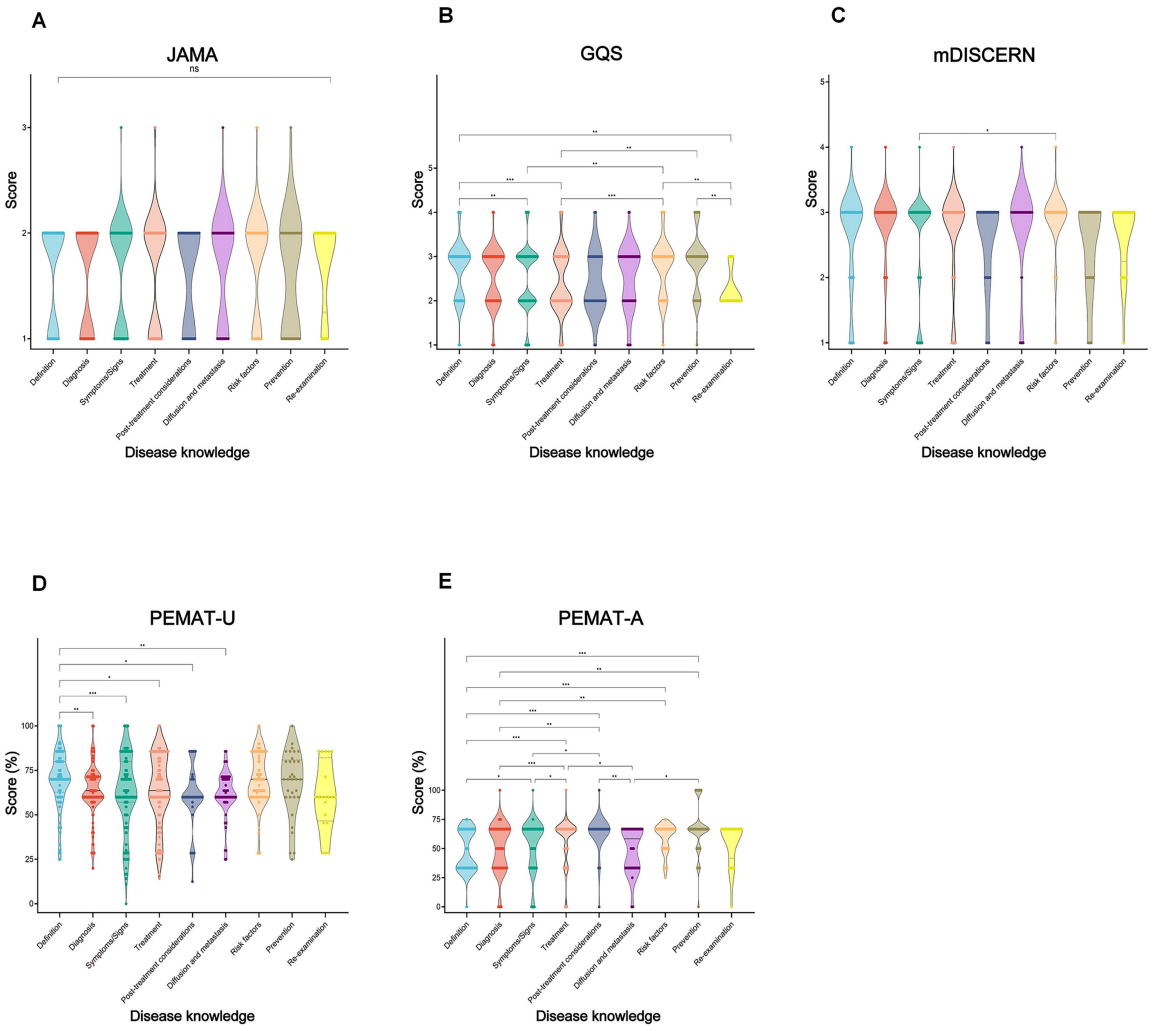
Comparative quality assessment of lung cancer short-videos with different disease knowledge. Violin plots showing quality evaluation of lung cancer short-videos multidisciplinary perspectives. **(A)** JAMA score; **(B)** GQS score; **(C)** mDISCERN score; **(D)** PEMAT-U; and **(E)** PEMAT-A. *p < 0.05, **p < 0.01, ***p < 0.001, ns: non-significant.

**Figure 8:**
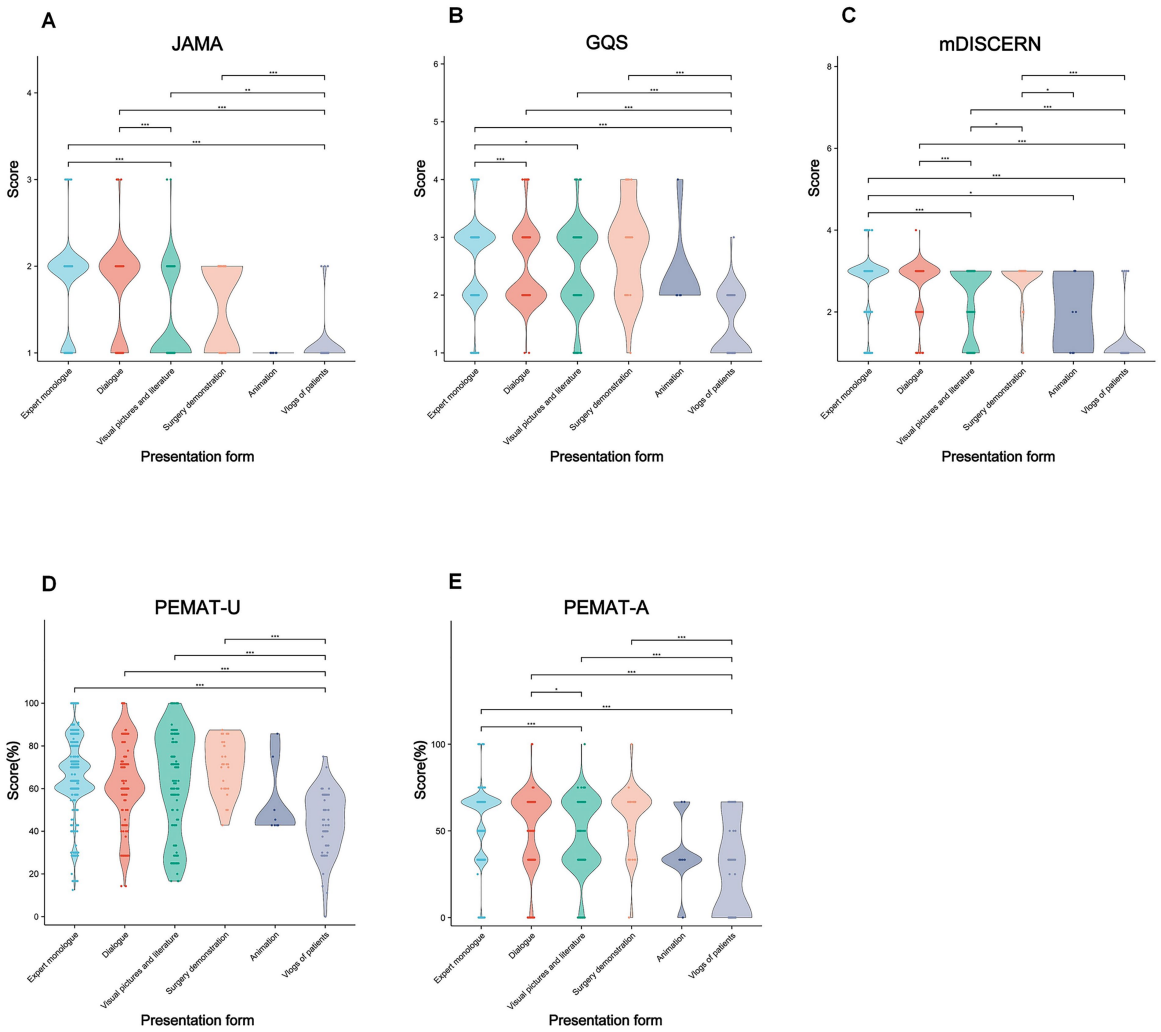
Comparative quality assessment of lung cancer short-videos with different presentation forms. Violin plots showing quality evaluation of lung cancer short-videos with different presentation form. **(A)** The JAMA score; **(B)** the GQS score; **(C)** the mDISCERN score; **(D)** PEMAT-U; and **(E)** PEMAT-A. *p < 0.05, **p < 0.01, ***p < 0.001, ns, non-significant.

As illustrated in Figures 5–8 and Supplementary Table S7, video source, content category, and presentation form significantly affected quality metrics (*η^2^* = 0.12–0.47, 95% CI [0.091, 0.542], *p* < 0.001). Professional sources outperformed lay creators across all indices (*η^2^* = 0.30–0.99, 95% CI [0.138, 0.998], *p* < 0.001–0.01). News agency videos achieved higher GQS and mDISCERN scores than physician-generated videos (*p* < 0.01–0.05). Among non-specialists, medical content creators scored higher than patients and their families (*p* < 0.001–0.01; Figure 5).

By content category, disease knowledge and outpatient-scene videos scored higher than personal-experience content (*p* < 0.001; Figure 6).

Within disease knowledge topics, prevention videos achieved the highest GQS, followed by definition and risk-factor content (*p* < 0.001–0.01; Figure 7). Risk factor videos achieved higher mDISCERN scores than symptom videos (*p* = 0.006). Definition-related videos yielded the highest PEMAT-U scores, whereas prevention achieved the highest PEMAT-A (*p* < 0.001). No significant differences were observed in JAMA across topics (*p* = 0.585).

Presentation form also influenced performance: expert monologs scored higher than animations and visual-literature videos across multiple indices (*p* < 0.001–0.05; Figure 8 and Supplementary Table S7).

### 3.4 Determinants of engagement

As summarized in Tables 2, 3, video source and disease topic significantly influenced engagement metrics (all *p* < 0.001). News agencies and hospitals achieved higher engagement than those from physicians (*p* < 0.001–0.05). News agencies achieved higher engagement than hospitals (*p* < 0.001). Patients and their families received more comments than physicians and medical creators (*η^2^* = 0.28–0.70, 95% CI [0.141, 0.85], *p* < 0.001–0.01).

**Table 2:** The widespread appeal of videos originating from various sources, contents and presentation forms.

Variables	Likes	Comments	Collections	Shares
Video source				
Physicians, median (IQR)	140 (29–800)	10 (2–46)	39 (8–232)	28 (5–168)
Medical content creators, median (IQR)	146 (19–563)	7 (1–34)	18 (2–131)	20 (2–118)
Patients and their families, median (IQR)	218 (24–3,096)	41 (6–788)	20 (3–305)	27 (4–227)
Hospitals, median (IQR)	12 (6–121)	2 (0–3)	2 (0–7)	3 (1–24)
News agencies, median (IQR)	426 (84–7,589)	22 (2–740)	73 (21–1,324)	118 (27–2,682)
Companies, median (Range)	622 (5–1239)^a^	57 (2–112)^a^	20 (1–38)^a^	17 (0–34)^a^
*P*-value	<0.001	<0.001	<0.001	<0.001
Video content				
Disease knowledge, median (IQR)	1881 (387.3–4,128)	124.5 (21.5–566)	566 (140.3–1,360)	315 (66.75–1,177)
Outpatient scenarios, median (IQR)	478.5 (57–3,154)	20 (3–283)	54 (13–553)	35 (5.3–539.8)
Personal experience, median (IQR)	76.5 (14.5–1,458)	21 (3–232)	15 (2–277)	11.5 (1.3–207.5)
*P*-value	0.78	0.08	0.31	0.33
Different disease knowledge				
Definition, median (IQR)	69 (13–424)	5 (1–31)	19 (3–82)	12 (1–79)
Diagnosis, median (IQR)	216 (40–1,020)	15 (3–80)	46 (9–251)	26 (6–131)
Symptoms, median (IQR)	229 (40–1,087)	9 (2–46)	39 (6–232)	25 (5–191)
Treatment, median (IQR)	114 (25–800)	11 (2–44)	45 (11–284)	33 (6–185)
Post-treatment considerations, median (IQR)	336 (37–1,470)	19 (1–96)	52 (14–471)	50 (8–391)
Diffusion and metastasis, median (IQR)	217 (31–2,418)	18 (3–269)	77 (14–537)	50 (5–749)
Risk factors, median (IQR)	87 (16–564)	4 (1–40)	15 (3–116)	23 (2–235)
Prevention, median (IQR)	394 (59–1,210)	13 (5–84)	54 (20–292)	66 (11–234)
Re-examination, median (IQR)	120 (20–2,346)	8 (0–29)	20 (11–190)	9 (5–94)
*P*-value	<0.001	<0.001	<0.001	<0.001
Video presentation form				
Expert monolog, median (IQR)	136 (29–733)	11 (2–47)	35 (7–197)	26 (5–165)
Dialog, median (IQR)	116 (26–1,333)	8 (2–44)	22 (8–285)	21 (5–187)
Visual pictures and literature, median (IQR)	200 (20–714)	7 (1–33)	54 (5–212)	35 (6–355)
Surgery demonstration, median (IQR)	215 (24–2,535)	32 (3–348)	48 (17–510)	60 (6–355)
Animation, median (IQR)	128 (9–12,808)	9 (0–528)	5 (0–769)	5 (2–2,458)
Vlogs of patients, median (IQR)	174 (13–2,758)	27 (3–508)	18 (3–302)	23 (2–225)
*P*-value	0.946	0.008	0.422	0.516

^a^For subgroups with *n* < 3, descriptive statistics were limited to median and range.

**Table 3:** Kruskal–Wallis test with a Dunn’s test results for lung cancer short-video popularity.

Comparisons		Test statistics	Standard error	Standard test statistics	Significance	Adj. sig
Likes						
Hospitals	Physicians	274.338	80.192	3.421	0.001	0.009
	Patients and their families	345.409	97.122	3.556	<0.001	0.006
News agencies	Hospitals	449.557	98.3	4.573	<0.001	<0.001
	Medical content creators	216.673	63.607	3.406	0.001	0.01
	Physicians	175.22	59.3	2.955	0.003	0.047
Definition	Diagnosis	−157.655	35.884	−4.393	<0.001	<0.001
	Symptoms	−174.036	33.28	−5.229	<0.001	<0.001
	Diffusion and metastasis	−195.969	54.172	−3.618	<0.001	0.011
	Prevention	−244.277	69.435	−3.518	<0.001	0.016
Comments						
Hospitals	Medical content creators	260.792	83.258	3.132	0.002	0.026
	Physicians	325.232	80.029	4.064	<0.001	<0.001
	News agencies	−440.907	98.101	−4.494	<0.001	<0.001
	Patients and their families	534.216	96.926	5.512	<0.001	<0.001
Patients and their families	Medical content creators	273.424	61.647	4.435	<0.001	<0.001
	Physicians	208.984	57.211	3.653	<0.001	0.004
Definition	Treatment	−109.866	32.948	−3.334	0.001	0.031
	Diagnosis	−154.799	35.811	−4.323	<0.001	0.001
	Diffusion and metastasis	−195.103	54.063	−3.609	<0.001	0.011
Visual pictures and literature	Vlogs of patients	−177.524	55.463	−3.201	0.001	0.021
Collections						
Hospitals	Medical content creators	272.863	83.404	3.272	0.001	0.016
	Patients and their families	330.693	97.095	3.406	<0.001	0.01
	Physicians	391.626	80.169	4.885	<0.001	<0.001
	News agencies	−520.875	98.272	−5.3	<0.001	<0.001
Medical content creators	Physicians	118.762	28.519	4.164	<0.001	<0.001
	News agencies	−248.011	63.59	−3.9	<0.001	0.001
Definition	Symptoms	−119.77	33.271	−3.6	<0.001	0.011
	Treatment	−147.987	33.006	−4.484	<0.001	<0.001
	Diagnosis	−155.599	35.874	−4.337	<0.001	0.001
	Diffusion and metastasis	−220.3	54.157	−4.068	<0.001	0.002
Risk factors	Diffusion and metastasis	197.209	58.876	3.35	0.001	0.029
Shares						
Hospitals	Physicians	259.4	80.125	3.237	0.001	0.018
News agencies	Hospitals	479.328	98.218	4.88	<0.001	<0.001
	Medical content creators	279.76	63.555	4.402	<0.001	<0.001
	Physicians	219.928	59.251	3.712	<0.001	0.003
Definition	Symptoms	−117.288	33.252	−3.527	<0.001	0.015
	Treatment	−130.074	32.988	−3.943	<0.001	0.003
	Diffusion and metastasis	−190.079	54.127	−3.512	<0.001	0.016
	Prevention	−228.77	69.377	−3.297	0.001	0.035

Engagement varied by topic: diffusion and metastasis videos achieved the highest levels of likes, comments, collections, and shares, whereas prevention videos obtained the greatest share frequency (*p* < 0.001–0.05).

Presentation formats significantly influenced comment volume (*p* = 0.008), vlogs of patients attracted more comments than visual pictures and literature (*p* < 0.05).

### 3.5 Correlation analysis

**Figure 9:**
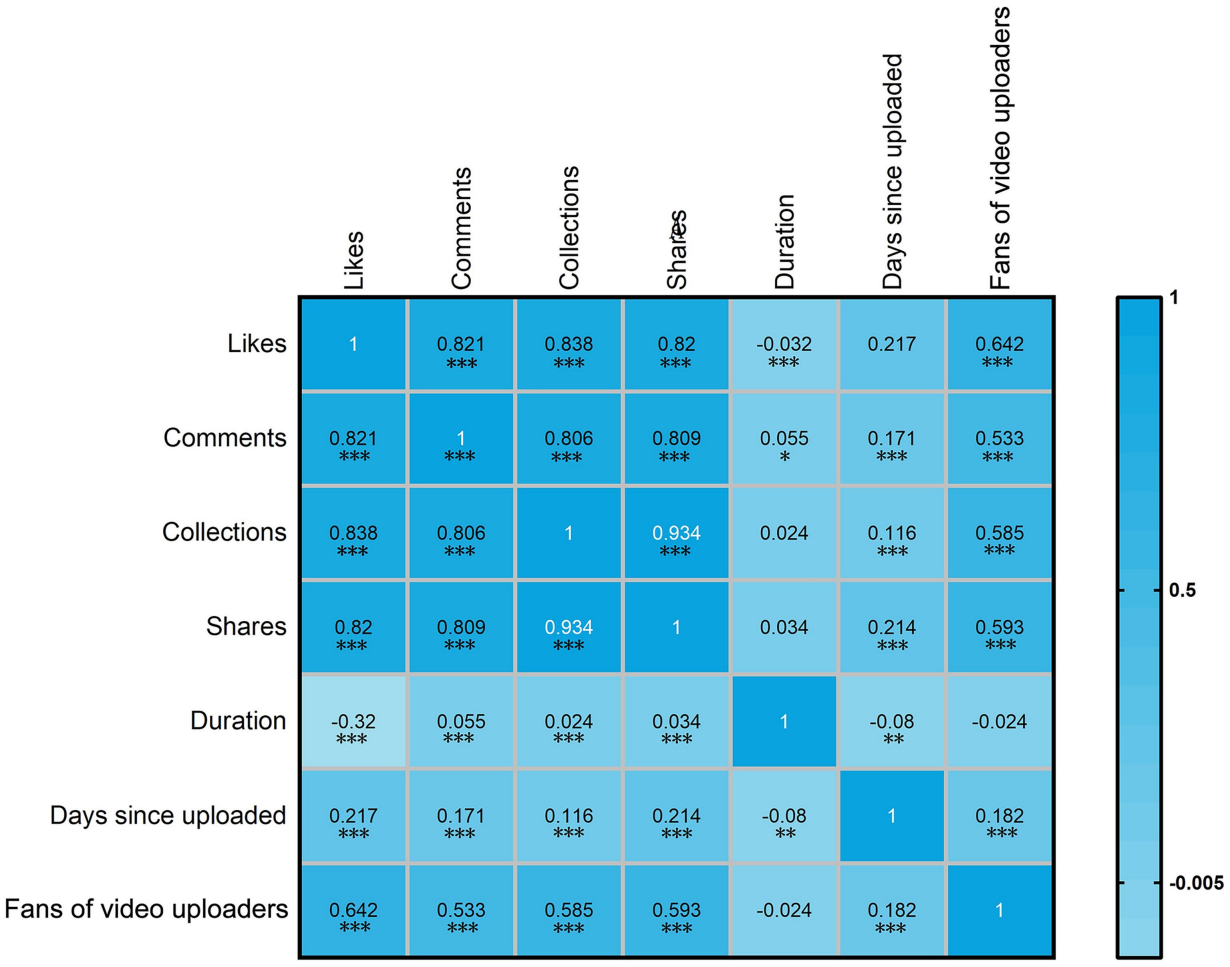
Spearman correlation heatmap of video engagement and general variables. This figure presents a Spearman correlation heatmap analyzing the relationships between video engagement metrics (likes, comments, collections, shares), temporal variables (duration, days since uploaded), and the number of fans of video uploaders. The heatmap uses a color gradient from −0.005 (light blue) to 1 (dark blue) to represent the strength and direction of correlations. Statistical significance is indicated by asterisks: *p < 0.05, **p < 0.01, ***p < 0.001.

Given the non-normal data distribution, Spearman’s correlation was applied. As shown in Figure 9 and Supplementary Table S8, likes, comments, collections, and shares were strongly correlated (*r* = 0.81–0.93, *p* < 0.001) and moderately correlated with follower count (*r* = 0.53–0.64, *p* < 0.001). Weak positive correlations were observed with days since upload (*r* = 0.12–0.22, *p* < 0.001).

**Figure 10:**
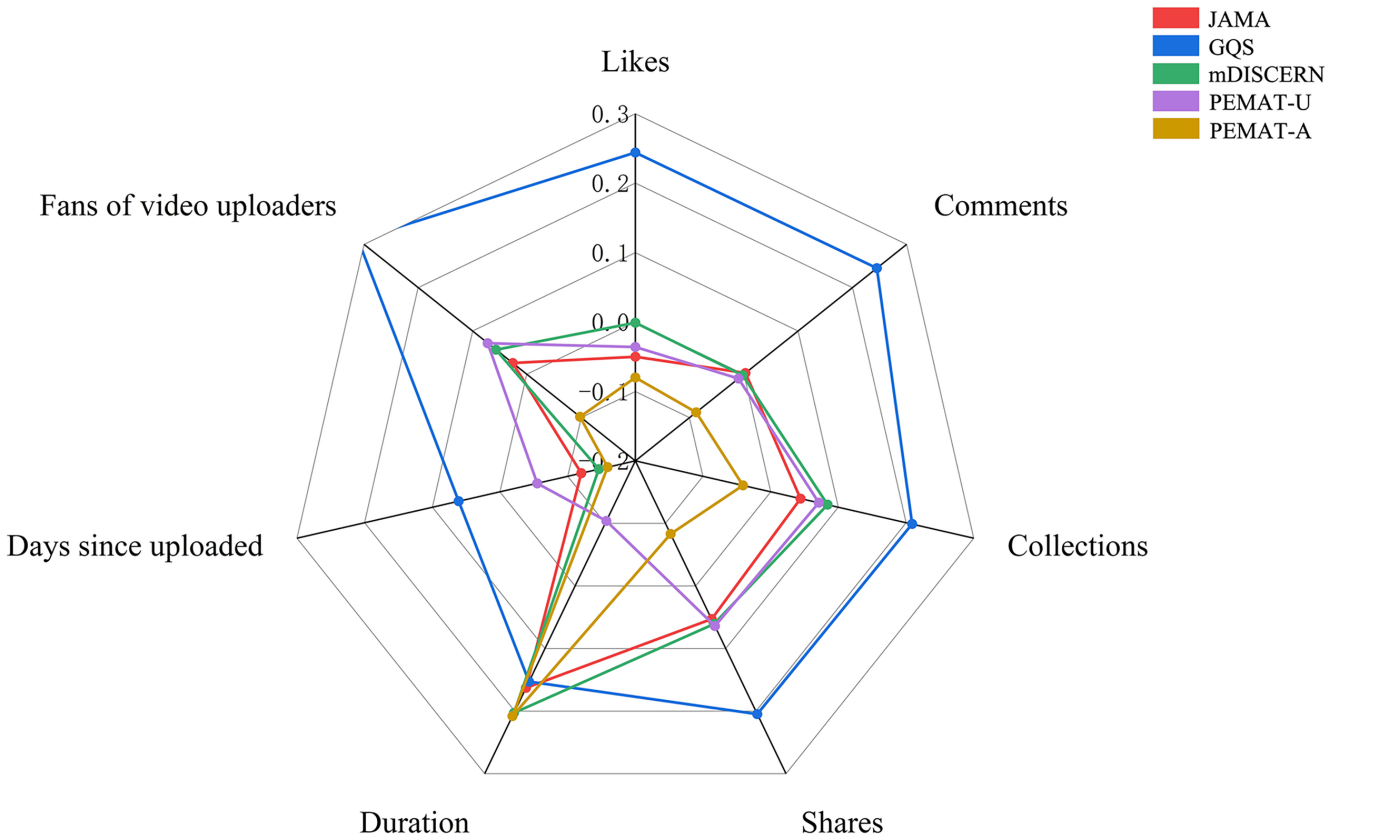
Radar chart of Spearman correlation coefficients between video quality metrics and video engagement variables. This radar chart illustrates the Spearman rank correlation coefficients between five video quality assessment tools (JAMA, GQS, mDISCERN, PEMAT-U, PEMAT-A) and seven video engagement metrics (Likes, Comments, Collections, Shares, Duration, Days since uploaded, Fans of video uploaders). Each axis represents an engagement metric, scaled from 0.0 to 0.3, with each video quality metric depicted by a colored line. The shape formed by each line provides a visual comparison of how strongly each quality assessment correlates with the various engagement indicators.

Among quality indicators, GQS correlated positively with all engagement metrics (*r* = 0.21–0.24, *p* < 0.001; Figure 10 and Supplementary Table S9). mDISCERN and PEMAT-U were weakly correlated with collections and shares (*r* = 0.06–0.08, *p* < 0.05). PEMAT-A showed weak negative correlations with likes, comments, and shares (*r* =,-0.09 to −0.08, *p* < 0.001). JAMA, mDISCERN, and PEMAT-A correlated positively with video duration (*r* = 0.16–0.21, *p* < 0.001) but negatively correlated with days since upload (*r* = −0.16 to −0.12, *p* < 0.001).

### 3.6 Summary of key findings

Lung cancer–related videos exhibited substantial heterogeneity in both informational quality and audience engagement. TikTok achieved the highest overall quality across all indices, while Kwai performed lowest. Videos from professionals—particularly news agencies and physicians—were more reliable, understandable, and actionable than those from laypersons. Disease-knowledge content, especially prevention, definition, and risk-factor topics, demonstrated superior quality, whereas personal-experience and metastasis videos scored lowest Expert monologs were the dominant and most effective presentation format. However, engagement did not correspond to informational quality: metastasis-related and patient-vlog videos generated greater interaction despite lower accuracy. Weak-to-moderate positive correlations between GQS and engagement metrics suggest that clearer and more actionable content may modestly enhance dissemination potential.

## 4 Discussion

### 4.1 Platform ecology and disparities in information quality

The cross-sectional analysis revealed significant disparities in the quality and dissemination performance of lung cancer–related content across China’s major short-video platforms. Overall, the information quality of these videos remained suboptimal, with high-quality videos accounting for only a limited proportion. This finding is consistent with previous studies on short-form videos related to gallstone, chronic obstructive pulmonary and premature ovarian failure (12, 26, 27).

Differences in platform ecosystems and moderation mechanisms largely determined the quality gap. TikTok demonstrated the highest video quality, likely due to its robust certification system for medical creators and stricter content review procedures, which provide institutional safeguards for professional participation (28, 29). Furthermore, its vast scale of health content—over 100 million videos and 500 billion views in the first half of 2023—creates a massive information pool that enables algorithms to more effectively identify and amplify higher-quality materials (30).

In contrast, Kwai received the lowest quality scores but the highest engagement. The platform hosts a higher proportion of non-professional creators, shorter duration, and visually oriented videos, reflecting an entertainment-driven dissemination logic. While possessing a dissemination advantage, this content struggles to maintain medical accuracy and evidence-based reliability.

Rednote ranked in between. Although approximately 85% of its videos were created by physicians, with 30% reenacting clinical scenarios, its moderation focus was more on verifying creator credentials rather than content validity. Consequently, these professionally staged videos were not effectively translated into accessible public knowledge, reducing their educational and dissemination impact. Collectively, these findings indicate that platform ecosystems define the upper boundary of information quality: TikTok operates as a professional moderation-oriented platform, Kwai is more algorithm- and interaction-oriented, and Rednote occupies a middle ground.

### 4.2 The impact of professional sources and content on video quality

Beyond platform-specific factors, video source, content theme, and presentation format were critical determinants of information quality. We found that videos uploaded by physicians and news agencies were of significantly higher overall quality than those from non-professional creators, aligning with findings from studies on hepatobiliary diseases and dry-eye syndrome (31–34). The superiority of physician-created videos stems from clinical experience and professional knowledge, enabling physicians to provide more practical guidance. News organizations, conversely, leverage collaborations with medical experts and professional editors to structure complex concepts into accessible narratives, enhancing understandability and usability while maintaining scientific integrity (30), Systematic review evidence further suggests that structured medical education videos effectively improve health literacy among both patients and the general public (35).

In terms of content type, disease knowledge videos, particularly those focusing on prevention, definitions, and risk factors, exhibited higher quality. This pattern is consistent with previous studies on thyroid disease, inflammatory myopathy, and frailty syndrome (36–38). Materials centered on knowledge transfer typically rely on evidence-based sources, such as clinical guidelines and randomized controlled trials (RCTs), thereby minimizing subjective or anecdotal bias and enhancing scientific accuracy. Preventive and risk-focused content offers greater actionability, whereas plain-language interpretations of definitions further improve understandability.

This finding carries important practical implications for public health literacy. In China and East Asian, public understanding of cancer screening principles and treatment risks remains limited, often leading to polarized behaviors of either neglecting early screening or pursuing unnecessary testing (39, 40). Promoting high-quality, evidence-based short videos on lung cancer can foster informed decision-making, encourage rational screening practices, and reduce the waste of medical resources.

Presentation format also shaped video quality. Expert monolog videos scored the highest, characterized by clear structure and logical coherence. They typically followed a “disease background–core knowledge–key takeaway” framework, integrating plain-language explanations of technical terms. This format enhanced systematic communication while preserving scientific rigor, reinforcing the observed advantage of professional sourcing.

Additionally, an overall improvement in video quality was observed over time. More recent uploads demonstrated greater completeness, evidence orientation, and practical value, suggesting that content moderation and platform governance are gradually being standardized (41, 42). A similar temporal trend was reported in YouTube studies on scoliosis, indicating that medical video quality on short-form platforms improves alongside the refinement of platform policies (43).

### 4.3 Drivers of video dissemination: source credibility, emotional resonance, and structural factors

Dissemination dynamics were influenced by source credibility. Institutional sources, such as news agencies and hospitals, received the highest overall engagement. This advantage is primarily attributed to their inherent authority, professional endorsement, and the trust conferred by official certification (44). In East Asian contexts, the public generally holds a high level of trust in official and professional institutions, a phenomenon validated during the COVID-19 pandemic information dissemination (45). High trust not only enhances the acceptance of health information but also mitigates the dissemination risk of false health claims (46).

Meanwhile, videos created by patients and their families generated more comments than those made by physicians. This difference suggests that first-person narratives foster empathy and peer identification. Research on cancer vlogs on YouTube similarly indicates that personal storytelling triggers stronger interactive responses (47). Comment sections often serve as venues for emotional exchange and mutual support rather than factual correction, which explains why high-quality videos from medical professionals tend to elicit fewer comments (48).

Content theme also played a significant role in dissemination. Videos discussing lung cancer dissemination and metastasis received the highest engagement. Given lung cancer’s status as a leading cause of cancer mortality in East Asia, public concern about disease progression gives this topic high dissemination potential (49). Such themes readily evoke high-arousal emotions like fear and anxiety, which can amplify user interaction (50). However, this mode of emotionally driven dissemination is double-edged: on one hand, it addresses the urgent information needs of patients regarding disease management; on the other, its anecdotal and emotional nature may exaggerate the condition, resulting in pessimistic perceptions of cancer and subsequently delaying screening, or conversely, causing over-diagnosis and over-treatment due to excessive anxiety (51–53).

Among structural factors, the creator follower count showed a moderate correlation with engagement, yet follower size alone cannot sustain visibility over time (54). Video duration displayed a bidirectional influence: longer videos were more likely to be shared and saved, reflecting higher perceived informational value and deeper cognitive engagement. Conversely, shorter videos tended to accumulate more likes by rapidly eliciting emotional reactions. Future health communication strategies should balance “depth of education” with “breadth of outreach” (55), for instance, by using short videos to attract broad attention and then guiding audiences toward more systematic health-learning content.

### 4.4 The non-linear relationship between information quality and dissemination

This study further revealed a partial non-linear relationship between information quality and dissemination. Highly engaging content could achieve extensive reach even with limited scientific rigor, a phenomenon consistent with analyses of short-form videos on thyroid and cardiovascular diseases (36, 56). We found that video quality, reliability, and understandability were weakly but positively correlated with saving and sharing behaviors, suggesting that audiences recognize such content as long-term reference material. However, the weak negative correlation between video actionability and engagement metrics implied that task-oriented content may lack emotional resonance, limiting their spread widely (57).

This non-linear pattern is largely driven by algorithmic mechanisms. Engagement metrics (likes, comments, saves, shares) were strongly intercorrelated (12, 58, 59). Recommendation algorithms typically treat early interactions, especially likes and comments, as positive feedback signals and assign them higher ranking weights. This creates an algorithmic reinforcement feedback loop that disproportionately favors emotionally salient or visually stimulating content (60). Prior studies on TikTok and Bilibili similarly observed that videos with higher engagement reached broader audiences (32, 36, 61).

This mechanism exert a dual impact on public health communication. On one hand, if high-quality health information can secure initial engagement, high-quality content that gains initial engagement can leverage algorithms for rapid dissemination and improved population-level health literacy. On the other hand, low-quality or misleading content can exploit the same mechanism to amplify misinformation, inducing unwarranted public anxiety or influencing clinical decision-making. Similar dynamics have been observed in studies of hypertension-related videos on TikTok, underscoring the challenges of communicating complex, actionable health information within emotion-driven recommendation environments (62). In the long term, this algorithmic bias may erode the educational function of health communication, exacerbate disparities in public health literacy, and undermine institutional efforts toward disease prevention and rational healthcare.

### 4.5 Practical implications, policy recommendations, and governance framework

The findings of this study provide multifaceted practical implications for optimizing the short-video health communication ecosystem, impacting content creators, platform governance, and public health policy.

#### 4.5.1 Implications for content creators

Medical professionals should balance scientific rigor with communication effectiveness. They should apply strategies from narrative communication and interactive design to enhance accessibility and engagement. Priority should be given to creating evidence-based medical knowledge content, translating complex oncological knowledge into actionable health guides (e.g., screening pathways, risk identification, treatment options) to promote public health literacy. Non-professional creators should clearly differentiate between “personal experiences” and “general medical advice” when sharing their stories, and reference authoritative guidelines or peer-reviewed sources to minimize misinformation risk.

#### 4.5.2 Recommendations for platform governance

This study has revealed a non-linear relationship between quality and engagement, whereby high-quality content does not always achieve wide dissemination, while emotionally charged materials are often algorithmically amplified. Platforms therefore bear a central responsibility for aligning engagement algorithms with information quality. Key governance measures include:

(a) Hybrid content vetting – Implementing a multilayered review system combining artificial intelligence (AI) detection with expert verification to flag potentially inaccurate or outdated information and reduce false-positive filtering.
(b) Structured metadata transparency – Making metadata fields such as creator credentials, evidence sources, update dates, and funding/conflict disclosures publicly available to improve algorithmic recognition and traceability of credible content.
(c) Versioning and periodic review – Collaborating with medical institutions to establish scheduled content audits, clearly marking or removing expired materials and publishing update logs and timestamps.
(d) Algorithmic accountability metrics – Evaluating platform performance using quantitative indicators, such as the reduction in misinformation exposure, increase in verified-source visibility, and improvements in audience comprehension scores. Through human–machine collaboration and transparent oversight, platforms can enhance both the precision and efficiency of health information dissemination.

#### 4.5.3 Implications for public health policy and ecosystem development

Public health authorities, academic institutions, professional societies, and news agencies should jointly cultivate a credible digital health information ecosystem.

First, news organizations and professional societies should act as a “bridge between evidence and the public,” enhancing information credibility and social impact through standard templates, expert verification, and tiered labeling systems.

Second, incentive programs for high-quality medical content can be introduced, promoting a “guideline-based content” classification system to guide the public toward reliable information sources.

Finally, media health literacy education should be integrated into community health education systems to improve the public’s overall information discernment capabilities. Regulatory bodies could require platforms to publish annual transparency reports, detailing medical information review baselines, data update frequencies, and ethical compliance processes to foster sustainable scientific communication and accountability.

### 4.6 Study limitations and constraints on interpretation

The findings of this study should be interpreted with the following limitations in mind.

First, limited platform scope: Only three dominant Chinese platforms (TikTok, Kwai, and Rednote) were included. Their algorithmic and audience structures differ from those of international platforms such as YouTube or Instagram, thereby limiting cross-cultural generalisability.

Second, temporal and design constraints: The cross-sectional design and single time-window sampling preclude analysis of longitudinal changes in video quality and algorithmic behavior. Correlations identified here should be regarded as associative rather than causal.

Third, measurement limitations: Engagement was assessed through common interaction metrics (likes, comments, shares, saves), which approximate attention rather than knowledge retention, attitudinal change, or behavioral outcomes.

Fourth, sampling bias: Analysis was restricted to the top 200 search results for each keyword, which are influenced by platform ranking and temporal volatility. Consequently, findings represent a snapshot of visible high-reach content rather than an exhaustive census.

### 4.7 Future research directions and academic perspectives

This study underscores both the transformative potential and the governance challenges of short-video platforms in public health communication. Future research can advance along three directions:

(1) Methodological innovation. Employ longitudinal tracking and pre-registered experimental or quasi-experimental designs to capture temporal changes in video quality and algorithmic dynamics.
(2) Broadened scope and comparative analysis. Future studies should include a wider range of diseases, languages, and international platforms to enable cross-cultural and cross-regulatory comparisons. Integrating platform transparency data with third-party audit reports would support global mapping of digital health communication patterns.
(3) Focus on real-world health outcomes and governance efficacy. Research should move beyond engagement metrics to develop validated tools for assessing educational and behavioral impact—such as gains in health literacy, screening uptake, or treatment adherence. Moreover, evaluating the performance of different ethical and regulatory frameworks will be essential for establishing scalable, responsible models of digital health governance.

## 5 Conclusion

This study systematically evaluated the quality and communication characteristics of lung cancer-related content on China’s three major short-video platforms (TikTok, Kwai, and Rednote). The findings reveal pronounced heterogeneity in health information quality and engagement dynamics and quantify a non-linear, quality–engagement relationship.

Despite generally suboptimal information quality, content produced by verified professionals proved more reliable, whereas emotionally driven, non-evidence-based materials achieved wider reach through affective resonance. This “quality-engagement paradox” highlights the inherent tension between emotional drivers and scientific rigor within the current algorithmic environment.

In the era of short-form video, advancing public health literacy requires not only sustained production of high-quality evidence-based content but also algorithmic and governance systems that integrate scientific validity into recommendation logic. Building an audience-centered, evidence-informed communication framework—supported by transparent data practices and multi-stakeholder collaboration—represents the key pathway to realizing the public-health potential of short-video platforms.

## Data Availability

The original contributions presented in the study are included in the article/Supplementary material, further inquiries can be directed to the corresponding authors.

## References

[1] Bray F Laversanne M Sung H Ferlay J Siegel RL Soerjomataram I . Global cancer statistics 2022: GLOBOCAN estimates of incidence and mortality worldwide for 36 cancers in 185 countries. CA Cancer J Clin. (2024) 74:229–63. doi: 10.3322/caac.21834, PMID: 38572751

[2] Chen W Zheng R Baade PD Zhang S Zeng H Bray F . Cancer statistics in China, 2015. CA Cancer J Clin. (2016) 66:115–32. doi: 10.3322/caac.21338, PMID: 26808342

[3] Islami F Torre LA Jemal A. Global trends of lung cancer mortality and smoking prevalence. Transl Lung Cancer Res. (2015) 4:327–38. doi: 10.3978/j.issn.2218-6751.2015.08.04, PMID: 26380174 PMC4549470

[4] Crosby D Bhatia S Brindle KM Coussens LM Dive C Emberton M . Early detection of cancer. Science. (2022) 375:eaay9040. doi: 10.1126/science.aay9040 35298272

[5] Sadate A Occean BV Beregi JP Hamard A Addala T de Forges H . Systematic review and meta-analysis on the impact of lung cancer screening by low-dose computed tomography. Eur J Cancer. (2020) 134:107–14. doi: 10.1016/j.ejca.2020.04.035 32502939

[6] Hsin-Hung C En-Kuei T Yun-Ju W Fu-Zong W. Impact of annual trend volume of low-dose computed tomography for lung cancer screening on overdiagnosis, overmanagement, and gender disparities. Cancer Imaging. (2024) 24:73. doi: 10.1186/s40644-024-00716-5 38867342 PMC11170916

[7] Wu FZ Huang YL Wu CC Tang EK Chen CS Mar GY . Assessment of selection criteria for low-dose lung screening CT among asian ethnic groups in Taiwan: from mass screening to specific risk-based screening for non-smoker lung cancer. Clin Lung Cancer. (2016) 17:e45–56. doi: 10.1016/j.cllc.2016.03.004, PMID: 27133540

[8] Qi Y Han J Lu X Wang Z Ren H Zhang X. A study on satisfaction evaluation of chinese mainstream short video platforms based on grounded theory and CRITIC-VIKOR. Heliyon. (2024) 10:e30050. doi: 10.1016/j.heliyon.2024.e30050, PMID: 38707463 PMC11068598

[9] Li Z Lin Y Zhang K Li R Ju M Chen Y . Hip fractures in chinese TikTok (douyin) short videos: an analysis of information quality, content and user comment attitudes. Front Public Health. (2025) 13:1563188. doi: 10.3389/fpubh.2025.1563188, PMID: 40342510 PMC12058786

[10] Li C Tham JS Ahmad GAH Hashim N Kim JN. Integrating the situational theory of problem solving and technology acceptance model to predict intention to practice health protective behavior for influenza-like illness among TikTok users: cross-sectional study. J Med Internet Res. (2025) 27:e73677. doi: 10.2196/73677, PMID: 40601922 PMC12268223

[11] Zhang Y Huang C Tong Y Teng Y Wan B Huang J . Quality assessment of spinal cord injury-related health information on short-form video platforms: cross-sectional content analysis of TikTok, kwai, and Bilibili. Digit Health. (2025) 11:20552076251374226. doi: 10.1177/20552076251374226, PMID: 40918080 PMC12409039

[12] Sun F Zheng S Wu J. Quality of information in gallstone disease videos on TikTok: cross-sectional study. J Med Internet Res. (2023) 25:e39162. doi: 10.2196/39162, PMID: 36753307 PMC9947761

[13] Shoemaker SJ Wolf MS Brach C. Development of the patient education materials assessment tool (PEMAT): a new measure of understandability and actionability for print and audiovisual patient information. Patient Educ Couns. (2014) 96:395–403. doi: 10.1016/j.pec.2014.05.027, PMID: 24973195 PMC5085258

[14] Silberg WM Lundberg GD Musacchio RA. Assessing, controlling, and assuring the quality of medical information on the internet: caveant lector et viewor—let the reader and viewer beware. JAMA. (1997) 277:1244–5. 9103351

[15] Morahan-Martin JM. How internet users find, evaluate, and use online health information: a cross-cultural review. Cyberpsychol Behav. (2004) 7:497–510. doi: 10.1089/cpb.2004.7.497, PMID: 15667044

[16] Lund BD Wang T Mannuru NR Nie B Shimray S Wang Z. ChatGPT and a new academic reality: artificial intelligence-written research papers and the ethics of large language models in scholarly publishing. J Assoc Inf Sci Technol. (2023) 74:570–81. doi: 10.1002/asi.24750

[17] Kwan JY Stocco F Scott DJA Bailey MA Coughlin PA. Assessment of internet-based information on statin therapy. Eur J Cardiovasc Nurs. (2024) 23:115–21. doi: 10.1093/eurjcn/zvad061, PMID: 37367216

[18] Chen Z Pan S Zuo S. TikTok and YouTube as sources of information on anal fissure: a comparative analysis. Front Public Health. (2022) 10:1000338. doi: 10.3389/fpubh.2022.1000338, PMID: 36407987 PMC9669434

[19] Zheng S Tong X Wan D Hu C Hu Q Ke Q. Quality and reliability of liver cancer-related short chinese videos on TikTok and bilibili: cross-sectional content analysis study. J Med Internet Res. (2023) 25:e47210. doi: 10.2196/47210, PMID: 37405825 PMC10357314

[20] He Z Wang Z Song Y Liu Y Kang L Fang X . The reliability and quality of short videos as a source of dietary guidance for inflammatory bowel disease: cross-sectional study. J Med Internet Res. (2023) 25:e41518. doi: 10.2196/41518, PMID: 36757757 PMC9951074

[21] Moon H Lee GH. Evaluation of korean-language COVID-19-related medical information on YouTube: cross-sectional infodemiology study. J Med Internet Res. (2020) 22:e20775. doi: 10.2196/20775, PMID: 32730221 PMC7425748

[22] Du RC Zhang Y Wang MH Lu NH Hu Y. TikTok and bilibili as sources of information on *helicobacter pylori* in China: a content and quality analysis. Helicobacter. (2023) 28:e13007. doi: 10.1111/hel.13007, PMID: 37452727

[23] Barlas T Ecem Avci D Cinici B Ozkilicaslan H Muhittin Yalcin M Eroglu Altinova A. The quality and reliability analysis of YouTube videos about insulin resistance. Int J Med Inform. (2023) 170:104960. doi: 10.1016/j.ijmedinf.2022.104960 36525801

[24] Zhang X Yang Y Shen YW Zhang KR Ma LT Ding C . Quality of online video resources concerning patient education for neck pain: a YouTube-based quality-control study. Front Public Health. (2022) 10:972348. doi: 10.3389/fpubh.2022.972348, PMID: 36211682 PMC9533122

[25] Akcay HC Kargu EC Seker N. Assessing the reliability and educational value of YouTube videos on computer-controlled local anesthesia in dentistry. PLoS One. (2025) 20:e0329291. doi: 10.1371/journal.pone.0329291 40773498 PMC12331094

[26] Song S Xue X Zhao YC Li J Zhu Q Zhao M. Short-video apps as a health information source for chronic obstructive pulmonary disease: information quality assessment of TikTok videos. J Med Internet Res. (2021) 23:e28318. doi: 10.2196/28318, PMID: 34931996 PMC8726035

[27] Xu R Ren Y Li X Su L Su J. The quality and reliability of short videos about premature ovarian failure on bilibili and TikTok: cross-sectional study. Digital Health. (2025) 11:20552076251351077. doi: 10.1177/20552076251351077, PMID: 40534890 PMC12174690

[28] Gong X Dong B Li L Shen D Rong Z. TikTok video as a health education source of information on heart failure in China: a content analysis. Front Public Health. (2023) 11:1315393. doi: 10.3389/fpubh.2023.1315393, PMID: 38146471 PMC10749320

[29] Liu Z Chen Y Lin Y Ai M Lian D Zhang Y . YouTube/Bilibili/TikTok videos as sources of medical information on laryngeal carcinoma: cross-sectional content analysis study. BMC Public Health. (2024) 24:1594. doi: 10.1186/s12889-024-19077-6 38877432 PMC11177428

[30] Gong X Chen M Ning L Zeng L Dong B. The quality of short videos as a source of coronary heart disease information on TikTok: cross-sectional study. JMIR Form Res. (2024) 8:e51513. doi: 10.2196/51513, PMID: 39226540 PMC11408897

[31] Huang M Wang J Wei J Zhang R Wang X Gan J . Assessing the quality of educational short videos on dry eye care: a cross-sectional study. Front Public Health. (2025) 13:1542278. doi: 10.3389/fpubh.2025.1542278, PMID: 40270739 PMC12014691

[32] Mao T Zhao X Jiang K Yang J Xie Q Fu J . Evaluation of TikTok videos on acute pancreatitis: content quality and reliability analysis. BMC Public Health. (2024) 24:1216. doi: 10.1186/s12889-024-18708-2, PMID: 38698404 PMC11067236

[33] Guan JL Xia SH Zhao K Feng LN Han YY Li JY . Videos in short-video sharing platforms as sources of information on colorectal polyps: cross-sectional content analysis study. J Med Internet Res. (2024) 26:e51655. doi: 10.2196/51655, PMID: 39470708 PMC11558218

[34] Dubovi I and Tabak I. Interactions between emotional and cognitive engagement with science on YouTube. (2021). Available at: https://journals.sagepub.com/doi/10.1177/0963662521990848?icid=int.sj-full-text.similar-articles.1 (Accessed August 3, 2025) 10.1177/0963662521990848 PMC8314998 33546572

[35] Langford A Loeb S. Perceived patient-provider communication quality and sociodemographic factors associated with watching health-related videos on YouTube: a cross-sectional analysis. J Med Internet Res. (2019) 21:e13512. doi: 10.2196/13512, PMID: 31102372 PMC6543799

[36] Chen Y Wang Q Huang X Zhang Y Li Y Ni T . The quality and reliability of short videos about thyroid nodules on BiliBili and TikTok: cross-sectional study. Digit Health. (2024) 10:20552076241288831. doi: 10.1177/20552076241288831 39381823 PMC11459542

[37] Kinugasa Y Adachi T Fukuki M Hirota Y Ishiga N Kato M . Factors affecting the willingness of nursing care staffs for cooperation with heart failure care and the role of internet video education. J Gen Fam Med. (2024) 25:19–27. doi: 10.1002/jgf2.658, PMID: 38239992 PMC10792320

[38] Ye L Ye Y Gao H. Effectiveness of social video platforms in promoting smoking cessation among youth: a content-specific analysis of smoking cessation topic videos on the social platform bilibili. Tob Induc Dis. (2023) 21:105. doi: 10.18332/tid/169662 37605770 PMC10405226

[39] He Z Ke Y. Challenge and future of cancer screening in China: insights from esophageal cancer screening practice. Chin J Cancer Res. (2023) 35:584–94. doi: 10.21147/j.issn.1000-9604.2023.06.03 38204451 PMC10774134

[40] Chan TKC Tan LWL van Dam RM Seow WJ. Cancer screening knowledge and behavior in a multi-ethnic Asian population: the Singapore community health study. Front Oncol. (2021) 11:684917. doi: 10.3389/fonc.2021.684917 34476210 PMC8406849

[41] Zhang R Zhang Z Jie H Guo Y Liu Y Yang Y . Analyzing dissemination, quality, and reliability of chinese brain tumor-related short videos on TikTok and bilibili: a cross-sectional study. Front Neurol. (2024) 15:1404038. doi: 10.3389/fneur.2024.1404038, PMID: 39494168 PMC11527622

[42] Kocamaz D Demircioğlu Karagöz A Atasavun Uysal S. YouTube videos as an information source about aerobic exercise in rehabilitation of lung cancer. Integr Cancer Ther. (2025) 24:15347354251331461. doi: 10.1177/15347354251331461, PMID: 40238487 PMC12034951

[43] Cinar C. A comparison of the quality and reliability of YouTube videos uploaded by healthcare professionals about scoliosis in the past decade. Cureus. (2023) 15:e44830. doi: 10.7759/cureus.44830, PMID: 37809187 PMC10559659

[44] Sun S Ge X Wen X Barrio F Zhu Y Liu J. The moderation of human characteristics in the control mechanisms of rumours in social media: the case of food rumours in China. Front Psychol. (2021) 12:782313. doi: 10.3389/fpsyg.2021.782313, PMID: 35111105 PMC8801587

[45] Liu H Chen Q Evans R. How official social media affected the infodemic among adults during the first wave of COVID-19 in China. Int J Environ Res Public Health. (2022) 19:6751. doi: 10.3390/ijerph19116751, PMID: 35682334 PMC9180041

[46] Lee SJ Lee CJ Hwang H. The impact of COVID-19 misinformation and trust in institutions on preventive behaviors. Health Educ Res. (2023) 38:95–105. doi: 10.1093/her/cyac038, PMID: 36564938

[47] Hale BJ Gonzales AL Richardson M. Vlogging cancer: predictors of social support in YouTube cancer vlogs. Cyberpsychol Behav Social Networking. (2018) 21:575–81. doi: 10.1089/cyber.2018.0176 30132690

[48] Ma Z Ma R Zhao X Wang X. Stories that engage the audience: an investigation of popular breast cancer narratives on social media. Telemat Inform. (2023) 85:102048. doi: 10.1016/j.tele.2023.102048

[49] Ding J Guo W Xue Q Cheng G Zhang L Wu D . Global and East Asia tracheal, bronchus, and lung cancer trend analysis from 1990 to 2021 and forecast trend from 2021 to 2035. Front Oncol. (2025) 15:151542067. doi: 10.3389/fonc.2025.1542067 PMC11960504 40171264

[50] Mazza M Piperis M Aasaithambi S Chauhan J Sagkriotis A Vieira C. Social media listening to understand the lived experience of individuals in Europe with metastatic breast cancer: a systematic search and content analysis study. Front Oncol. (2022) 12:863641. doi: 10.3389/fonc.2022.863641, PMID: 35719996 PMC9205394

[51] Cancer overdiagnosis: a challenge in the era of screening. J Natl Cancer Cent. (2022) 2:235–42. doi: 10.1016/j.jncc.2022.08.005, PMID: 36568283 PMC9784987

[52] Loeb Stacy Langford Aisha T Bragg Marie A Sherman Robert Chan June M. Cancer misinformation on social media. CA Cancer J Clin. 2024 74, 453–464. doi: 10.3322/caac.21857 38896503 PMC11648589

[53] Hung YC Lin Y Wu FZ. Editorial: advancements and challenges in lung cancer screening, diagnosis, and management. Diagnostics. (2025) 15:835. doi: 10.3390/diagnostics15070835, PMID: 40218185 PMC11988577

[54] Wang J Xu K Wu J Liang W Qiu W Wang S. Evaluating the content and quality of videos related to hypertrophic scarring on TikTok in China: cross-sectional study. JMIR Infodemiol. (2025) 5:e64792. doi: 10.2196/64792 PMC12076032 40300161

[55] Cheng Z Li Y. Like, comment, and share on TikTok: exploring the effect of sentiment and second-person view on the user engagement with TikTok news videos. Soc Sci Comput Rev. (2024) 42:201–23. doi: 10.1177/08944393231178603

[56] Li Q Jin L Shi K Zheng X. Short-video platforms as sources of atherosclerosis information: a cross-sectional content analysis. Medicine (Baltimore). (2025) 104:e45006. doi: 10.1097/MD.0000000000045006, PMID: 41054099 PMC12499849

[57] Metzler H Garcia D. Social drivers and algorithmic mechanisms on digital media. Perspect Psychol Sci. (2024) 19:735–48. doi: 10.1177/17456916231185057, PMID: 37466493 PMC11373151

[58] Yu JS Carr JB Thomas J Kostas J Wang Z Khilnani T . Trends in patient, physician, and public perception of ulnar collateral ligament reconstruction using social media analytics. Orthop J Sports Med. (2021) 9:2325967121990052. doi: 10.1177/2325967121990052, PMID: 34250162 PMC8239339

[59] Li A Xing Q Zhang Y Zhan W Zhu S Lou L . Evaluation of the information quality related to osteoporosis on TikTok. BMC Public Health. (2024) 24:2880. doi: 10.1186/s12889-024-20375-2 39425064 PMC11490150

[60] Yang C. Bias in short-video recommender systems: user-centric evaluation on TikTok. Carolina Digital Repository. (2022). doi: 10.17615/ttth-sg62

[61] Tu J Zhang C Zhang H Liang L He J. Evaluating the reliability and quality of knee osteoarthritis educational content on TikTok and bilibili: a cross-sectional content analysis. Digital Health. (2025) 11:20552076251366390. doi: 10.1177/20552076251366390, PMID: 40808711 PMC12344336

[62] Wu J Wu G Che X Guo J. The quality and reliability of short videos about hypertension on TikTok: a cross-sectional study. Sci Rep. (2025) 15:25042. doi: 10.1038/s41598-025-08680-1 40646027 PMC12254506

