# Supplementary material for: Quality, reliability, and dissemination of lung cancer information on short-video platforms in China: a cross-platform content analysis of TikTok, Kwai, and Rednote
Source: Front Public Health. 2025 Dec 1;13:1683561. doi: 10.3389/fpubh.2025.1683561 (PMC12702867; doi:10.3389/fpubh.2025.1683561)
Supplement: Supplementary file 2 [file Data_Sheet_2.docx]

S5-Table 2. The sources and contents of the Lung cancer–related videos.

| Variable | Total  (N=1288), n (100%) | TikTok  (n=454), n (35.25%) | Kwai  (n=428), n (33.23%) | rednote  (n=406), n (31.52%) | *P* value |
| --- | --- | --- | --- | --- | --- |
| **Video source** |  |  |  |  | <0.001 |
| Physicians | 973(75.54) | 386(85.02) | 242(56.54) | 345(84.98) |  |
| Medical content creators | 206(15.99) | 18(3.96) | 147(34.35) | 41(10.1) |  |
| Patients and their families | 44(3.42) | 15(3.3) | 13(3.04) | 16(3.94) |  |
| Hospitals | 22(1.71) | 19(4.19) | 2(0.47) | 1(0.25) |  |
| News agencies | 41(3.18) | 15(3.3) | 24(5.61) | 2(0.49) |  |
| Companies | 2(0.16) | 1(0.22) | 0(0) | 1(0.25) |  |
| **Video content** |  |  |  |  | <0.001 |
| Disease knowledge | 1022(79.35) | 379(83.48) | 346(80.84) | 297(73.15) |  |
| Outpatient scenarios | 198(15.37) | 57(12.56) | 59(13.79) | 82(20.2) |  |
| Personal experience | 68(5.28) | 18(3.96) | 23(5.37) | 27(6.65) |  |
| **Different disease knowledge** |  |  |  |  | 0.288 |
| Definition | 220(17.08) | 67(14.76) | 86(20.09) | 67(16.5) |  |
| Diagnosis | 210(16.3) | 97(21.37) | 48(11.21) | 65(16.01) |  |
| Symptoms | 289(22.44) | 100(22.03) | 129(30.14) | 60(14.78) |  |
| Treatment | 300(23.29) | 83(18.28) | 82(19.16) | 135(33.25) |  |
| Post-treatment considerations | 37(2.87) | 5(1.1) | 13(3.04) | 19(4.68) |  |
| Diffusion and metastasis | 60(4.66) | 29(6.39) | 12(2.8) | 19(4.68) |  |
| Risk factors | 119(9.24) | 65(14.32) | 27(6.31) | 27(6.65) |  |
| Prevention | 33(2.56) | 5(1.1) | 23(5.37) | 5(1.23) |  |
| Re-examination | 20(1.55) | 3(0.66) | 8(1.87) | 9(2.22) |  |
| **Video presentation form** |  |  |  |  | 0.002 |
| Expert monologue | 820(63.66) | 316(69.6) | 245(57.24) | 259(63.79) |  |
| Dialogue | 187(14.52) | 47(10.35) | 62(14.49) | 78(19.21) |  |
| Visual pictures and literature | 186(14.44) | 56(12.33) | 93(21.73) | 37(9.11) |  |
| Surgery demonstration | 28(2.17) | 15(3.3) | 6(1.4) | 7(1.72) |  |
| Animation | 8(0.62) | 0(0) | 7(1.64) | 1(0.25) |  |
| Vlogs of patients | 59(4.58) | 20(4.41) | 15(3.5) | 24(5.91) |  |

S6 Table. The general characteristics and scores of the Lung cancer–related videos.

| Scale/Score | Total  (N=1288), n (100%) | TikTok(n=454), n(35.25%) | Kwai(n=428), n(33.23%) | rednote(n=406), n(31.52%) |
| --- | --- | --- | --- | --- |
| **JAMA** |  |  |  |  |
| 0-1 | 476（36.96%） | 128（28.19%） | 213（49.77%） | 135（33.25%） |
| 2-3 | 812（63.04%） | 326（71.81%） | 215（50.23） | 271（66.75%） |
| 4 | 0（0%） | 0（0%） | 0（0%） | 0（0%） |
| **GQS** |  |  |  |  |
| 1-2 | 572（44.41%） | 40（8.81%） | 181（42.29%） | 351（86.45%） |
| 3 | 644（50%） | 382（84.14%） | 212（49.53%） | 50（12.32%） |
| 4-5 | 40（5.59%） | 32（7.05%） | 35（8.17%） | 5（1.23%） |
| **modified DISCERN** |  |  |  |  |
| 1-2 | 287（22.29%） | 43（9.47%） | 173（40.42%） | 71（17.49%） |
| 3 | 988（76.71%） | 408（89.87%） | 247（57.71%） | 333（82.02%） |
| 4-5 | 13（1.01%） | 3（0.66%） | 8（1.87%） | 2（0.49%） |
| **PEMAT-U** |  |  |  |  |
| 0-59% | 267（20.73%） | 36（7.93%） | 183（42.76%） | 48（11.82%） |
| 60%-79% | 697（54.11%） | 315（69.38%） | 151（35.28%） | 305（75.12%） |
| 80%-100% | 324（25.16%） | 103（22.69%） | 120（28.04%） | 101（24.88%） |
| **PEMAT-A** |  |  |  |  |
| 0-59% | 528（40.99%） | 146（32.16%） | 261（60.98%） | 121（29.8%） |
| 60%-79% | 750（58.23%） | 307（67.62%） | 158（36.92%） | 285（70.2%） |
| 80%-100% | 10（0.78%） | 1（0.22%） | 9（2.1%） | 0（0%） |

S7-Table 3-Fig.4-7. The evaluation from different sources with different contents and different presentation forms.

| Variables | JAMA | GQS | modified DISCERN | PEMAT–U | PEMAT–A |
| --- | --- | --- | --- | --- | --- |
| **Video source** |  |  |  |  |  |
| Physicians, median (IQR) | 2(2-2) | 3(2-3) | 3(3-3) | 70%(60%-80%) | 66.67%(50%-66.67%) |
| Medical content creators, median (IQR) | 1(1-1) | 2(2-3) | 2(1-3) | 57.14%(33.33%-70%) | 33.33%(33.33%-66.67%) |
| Patients and their families, median (IQR) | 1(2-2) | 2(1-2) | 1(1-1) | 40%(28.57%-50%) | 25%(0-66.67%) |
| Hospitals, median (IQR) | 2(2-3) | 3(3-3) | 3(3-3) | 70%(63.64%-72.73%) | 58.34%(50%-66.67%) |
| News agencies, median (IQR) | 2(1-2) | 3(3-4) | 3(2.5-3) | 75%(70.71%-85.71%) | 66.67%(41.67%-66.67%) |
| Companies, median (IQR) | 1(1-1) | 2(2-2) | 2(1-3) | 50%(42.86%-57.14%) | 16.67%(0-33.33%) |
| *P* value | <0.001 | <0.001 | <0.001 | <0.001 | <0.001 |
| **Video content** |  |  |  |  |  |
| Disease knowledge, median (IQR) | 2(1-2) | 3(2-3) | 3(3-3) | 70.00%(60.00%-80.00%) | 66.67%(50.00%-66.67%) |
| Outpatient scenarios, median (IQR) | 2(1-2) | 2(2-3) | 3(3-3) | 60.00%(57.14%-83.77) | 66.67%(33.33%-66.67%) |
| Personal experience, median (IQR) | 2(1-1) | 2(1-2) | 1(1-1) | 40.00%(28.57%-50.00%) | 33.33%(0-66.67%) |
| *P* value | <0.001 | <0.001 | <0.001 | <0.001 | <0.001 |
| **Different disease knowledge** |  |  |  |  |  |
| Definition, median (IQR) | 2(1-2) | 3(2-3) | 3(3-3) | 70.00%(63.64%-80.00%) | 50.00%(33.33%-66.67%) |
| Diagnosis,median (IQR) | 2(1-2) | 3(2-3) | 3(3-3) | 60.00%(60.00%-70.00%) | 66.67%(50.00%-66.67%) |
| Symptoms, median (IQR) | 2(1-2) | 3(2-3) | 3(3-3) | 60.00%(57.14%-80.00%) | 66.67%(33.33%-66.67%) |
| Treatment, median (IQR) | 2(1-2) | 2(2-3) | 3(3-3) | 63.64%(60.00%-85.71%) | 66.67%(50.00%-66.67%) |
| Post-treatment considerations, median (IQR) | 2(1-2) | 2(2-3) | 3(2-3) | 60.00%(60.00%-70.00%) | 66.67%(66.67%-66.67%) |
| Diffusion and metastasis, median (IQR) | 2(1-2) | 3(2-3) | 3(3-3) | 68.58%(60.00%-70.00%) | 66.67%(33.33%-66.67%) |
| Risk factors, median (IQR) | 2(1-2) | 3(2-3) | 3(3-3) | 63.64%(60.00%-73.87%) | 66.67%(50.00%-66.67%) |
| Prevention, median (IQR) | 2(1-2) | 3(3-3) | 3(2-3) | 70.00%(60.00-80.91%) | 66.67%(66.67%-66.67%) |
| Re-examination, median (IQR) | 2(1.5-2) | 2(2-2) | 3(2.5-3) | 60.00%(47.73%-78.57%) | 66.67%(50.00%-66.67%) |
| *P* value | 0.585 | <0.001 | 0.006 | <0.001 | <0.001 |
| **Video presentation form** |  |  |  |  |  |
| Expert monologue, median (IQR) | 2(1-2) | 3(2-3) | 3(3-3) | 63.64%(60.00%-72.73%) | 66.67%(50.00%-66.67%) |
| Dialogue, median (IQR) | 2(1-2) | 2(2-3) | 3(3-3) | 60.00%(60.00%-85.71%) | 66.67%(33.33%-66.67%) |
| Visual pictures and literature, median (IQR) | 1(1-2) | 3(2-3) | 3(2-3) | 70.00%(33.33%-85.71%) | 66.67%(33.33%-66.67%) |
| Surgery demonstration, median (IQR) | 2(1-2) | 3(2-3) | 3(3-3) | 71.43%(60.00%-81.82%) | 66.67%(50.00%-66.67%) |
| Animation, median (IQR) | 1(1-1) | 2(2-2) | 2(1-3) | 44.16%(42.86%-62.50%) | 33.33%(33.33%-50.00%) |
| Vlogs of patients, median (IQR) | 1(1-1) | 2(1-2) | 1(1-1) | 42.86%(28.57%-52.28%) | 33.33%(0-66.67%) |
| *P* value | <0.001 | <0.001 | <0.001 | <0.001 | <0.001 |

S8-Table 6-Fig.8. The correlation analysis between video variables.

| **Variables** | **Likes** | **Comments** | **Collections** | **Shares** | **Duration** | **Days since uploaded** | **Fans** |
| --- | --- | --- | --- | --- | --- | --- | --- |
| **Likes** |  |  |  |  |  |  |  |
| ρ | 1 | 0.821 | 0.838 | 0.82 | -0.32 | 0.217 | 0.642 |
| *P* value | __^a^ | <0.001 | <0.001 | <0.001 | <0.001 | 0.247 | <0.001 |
| **Comments** |  |  |  |  |  |  |  |
| ρ | 0.821 | 1 | 0.806 | 0.809 | 0.055 | 0.171 | 0.533 |
| *P* value | <0.001 | __^a^ | <0.001 | <0.001 | 0.049 | <0.001 | <0.001 |
| **Collections** |  |  |  |  |  |  |  |
| ρ | 0.838 | 0.806 | 1 | 0.934 | 0.024 | 0.116 | 0.585 |
| *P* value | <0.001 | <0.001 | __^a^ | <0.001 | 0.381 | <0.001 | <0.001 |
| **Shares** |  |  |  |  |  |  |  |
| ρ | 0.820 | 0.809 | 0.934 | 1 | 0.034 | 0.214 | 0.593 |
| *P* value | <0.001 | <0.001 | <0.001 | __^a^ | 0.226 | <0.001 | <0.001 |
| **Duration** |  |  |  |  |  |  |  |
| ρ | -0.032 | 0.055 | 0.024 | 0.034 | 1 | -0.080 | -0.024 |
| *P* value | <0.001 | <0.001 | <0.001 | <0.001 | __^a^ | 0.004 | 0.397 |
| **Days since uploaded** |  |  |  |  |  |  |  |
| ρ | 0.217 | 0.171 | 0.116 | 0.214 | -0.080 | 1 | 0.182 |
| *P* value | <0.001 | <0.001 | <0.001 | <0.001 | 0.004 | __^a^ | <0.001 |
| **Fans** |  |  |  |  |  |  |  |
| ρ | 0.642 | 0.533 | 0.585 | 0.593 | -0.024 | 0.182 | 1 |
| *P* value | <0.001 | <0.001 | <0.001 | <0.001 | 0.397 | <0.001 | __^a^ |

^a^Not applicable.

S9Table 7-Fig.9. The correlation analysis between video variables and the video quality.

| Variables | JAMA^a^ | GQS^b^ | modified DISCERN | PEMAT–U | PEMAT–A |
| --- | --- | --- | --- | --- | --- |
| **Likes** |  |  |  |  |  |
| ρ | -0.050 | 0.244 | -0.001 | -0.036 | -0.080 |
| *P* value | 0.75 | <0.001 | 0.983 | 0.219 | 0.006 |
| **Comments** |  |  |  |  |  |
| ρ | 0.003 | 0.245 | -0.002 | -0.010 | -0.088 |
| *P* value | 0.901 | <0.001 | 0.957 | 0.742 | 0.002 |
| **Collections** |  |  |  |  |  |
| ρ | 0.044 | 0.209 | 0.084 | 0.071 | -0.041 |
| *P* value | 0.118 | <0.001 | 0.002 | 0.015 | 0.158 |
| **Shares** |  |  |  |  |  |
| ρ | 0.053 | 0.205 | 0.061 | 0.064 | -0.083 |
| *P* value | 0.059 | <0.001 | 0.030 | 0.027 | 0.004 |
| **Duration** |  |  |  |  |  |
| ρ | 0.163 | 0.153 | 0.203 | -0.104 | 0.208 |
| *P* value | <0.001 | 0.342 | <0.001 | <0.001 | <0.001 |
| **Days since uploaded** |  |  |  |  |  |
| ρ | -0.120 | 0.061 | -0.146 | -0.055 | -0.159 |
| *P* value | <0.001 | 0.052 | <0.001 | 0.058 | <0.001 |
| **Fans** |  |  |  |  |  |
| ρ | 0.026 | 0.311 | 0.057 | 0.072 | -0.098 |
| *P* value | 0.356 | <0.001 | 0.041 | 0.013 | <0.001 |

^a^JAMA: Journal of American Medical Association.

^b^GQS: Global Quality Scale.
